# Haematological Profile and Antibiotic Resistance of Bacteria Responsible for Enteric Infections Isolated From Patients Suffering From Malaria and Enteric Infections on Consultation at the Dschang Regional Hospital

**DOI:** 10.1155/2024/3383995

**Published:** 2024-10-25

**Authors:** Roland Y. Ngai, Wiliane J. T. Marbou, Armelle T. Mbaveng, Victor Kuete

**Affiliations:** Department of Biochemistry, Faculty of Science, University of Dschang, Cameroon

**Keywords:** antibiotic resistance, bacteria, enteric infections, haematological profile, malaria

## Abstract

Malarial and bacterial coinfections in low-income countries are a serious cause of morbidity and mortality, necessitating coadministration of antibiotics and antimalarials. This study investigated the relationship between malaria infection and bacterial drug resistance in malaria and nonmalaria patients on consultation at the Dschang Regional Hospital. A follow-up study was carried out from October 2020 to December 2021 on 127 malaria and 174 nonmalaria patients having enteric infections. Clinical and haematological parameters were measured using standard methods. CD4 and CD8 cells were determined using flow cytometry. Enteric bacteria pathogens were isolated from stool, and antimicrobial and antimalarial profiles were determined using agar diffusion and microdilution methods, respectively. Significant reduction of RBCs, WBCs, CD4, CD8, granulocytes, monocytes and platelets was seen in coinfected patients compared to monoinfected participants (*p* ≤ 0.0491). *E. coli* was the main pathogenic bacteria isolated from the digestive tract of coinfected patients (40.63%) and monoinfected patients (59.37%). *E. coli* showed a high level of resistance to AMX (57.69%) and CDA (61.54%) in coinfected patients compared to 55.26% and 41.67%, respectively, in monoinfected patients. Quinine (53[50.00%]; 6[42.86%]) presented a minimal inhibitory concentration (MIC) of 32 μg/mL on the bacteria isolates from coinfected and monoinfected patients, respectively, while Artemether 89 (83.96%), Maloxine 5 (3.94%) and Surquina 250 (39.37%) presented a MIC of 64 μg/mL on bacterial isolates of coinfected and monoinfected patients. *E. coli* showed high resistance against AKI (45.93%), AMX (43.75%) and ERY (59.37%) in malaria patients who were under antimalarial drugs compared to malaria patients who were not under malaria drugs (29.68%, 34.37% and 32.81%, respectively). This study highlights that antimalarial drugs might certainly have an influence on the acquisition and emergence of bacterial resistance in the case of malaria bacterial coinfection, and therefore, adequate management and planning effective control programmes might certainly go a long way to reduce the rate of morbidity and mortality.

## 1. Introduction

In spite of an increase in control measures, malaria still remains a major public health problem in sub-Saharan Africa. In Cameroon, each year, an estimated 41% of the population had at least one episode of malaria with children under 5 years and pregnant women mostly affected [1]. Malaria eradication has shown its numerous limits due to the limitations of implementation of effective control measures, thus making the initial decrease in the burden of the disease to have slowed down and even completely come to a stop in some countries, and consequently, efforts to control transmission are jeopardized due to the spread of insecticide resistance in mosquitoes [2, 3]. Indeed, it is well known that malaria infection is a parasitosis quite frequently accompanied by haematological disturbance [4]. Surveys have proven the utmost presence of a majority of the plasmodium parasites, through microscopy and molecular analysis in the spleen and bone marrow. The presence of these parasites in the bone marrow raises questions about erythropoiesis, processes leading to the production of red blood cells from the haematopoietic stem cells [5, 6]. Malaria infection has devastating consequences as a result of the weakened immune system due to the effects of malaria antigen-induced polarization of T cells that cause immunodepression [7], leading to opportunistic infections among which bacterial infections consisting of both Gram-negative and Gram-positive bacteria can then profit [8]. These endemic infections at times are predicted through the measurement of haematological parameters [9, 10]. The rate of gastrointestinal infections has been favoured by harsh climatic and socioeconomic conditions in tropical and subtropical regions. In low-income countries, morbidity and mortality caused by enteric infections are alarmingly increasing [11]. Nevertheless, antimicrobials have constantly been employed for their partial or complete eradication and their misuse has greatly resulted to the phenomenon of resistance across geographical areas and countries of the world [12, 13].

The antibiotic resistance of several bacteria from various environmental and human sources is an increasing public health emergency since infections from resistant phenotypes are more complex and costly [14–16]. Bacterial and malaria coinfections are common in malaria endemic countries and thus necessitate coadministration of antibiotics and antimalarials [17]. A study carried out in Switzerland revealed that *P. falciparum* was the predominant species (59%) during bacterial coinfection with a high risk of bacterial infection in children having acute malaria [18]. However, studies by Sallares and Cunnington have shown that quinine, an antimalarial drug, was able to spontaneously get rid of coinfected individuals of bacterial infections without additional treatment [19–21]. This may indicate that antimalarial drugs could be nonspecific and have antibacterial activity. Unfortunately, very little is known about whether the antimalarial drugs used to treat malaria have antibacterial activity and the consequence of this activity on bacteria susceptibility to antibiotics in patients with enteric infections. This study aimed at investigating the relationship between malaria infection and bacterial drug resistance in order to ensure a better management of patients with enteric disorders, thus contributing to the fight against infectious diseases in a bit of reducing the morbidity and mortality rates, and to concretely determine the aetiologies of these coinfections and their associations with haematological parameters of patients on consultation at the Dschang Regional Hospital.

## 2. Material and Methods

### 2.1. Study Site

This study was carried out at the Dschang Regional Hospital of the Menoua Division, West Region of Cameroon. The Dschang Regional Hospital is situated in the West Region of Cameroon, Menoua Division, more precisely at the heart of Dschang town. It is located between latitude 5°25′-5°-30′ North and longitude 10°-10°-5 [22].

### 2.2. Study Design

A follow-up study was conducted from October 2020 to December 2021 at the Dschang Regional Hospital of the Menoua Division, West Region, Cameroon. Patients recruited in this study were those with malaria and/or enteric infections with a history of frequent intake of malaria drugs or not, as well as those suffering only from enteric infection.

### 2.3. Study Population

This study included participants having malaria and enteric infections on consultation at the Dschang Regional Hospital irrespective of sex and age. A total of 301 volunteers were recruited for this study, consisting of 127 malaria patients, and related enteric infections and 174 nonmalarial patients with enteric infections were recruited for this study.

### 2.4. Ethical Approval

To minimize the problem regarding researchers' conflicts of interest and to ensure the adequate protection of the rights and welfare of human participants, an ethical clearance was obtained from the Ethical Committee CRERSH-OUEST (Reference no. 523/30/06/2023/CE/CRERSH-OU) before the onset of this study, and research authorization was obtained from the Dschang Regional Hospital, before getting into contact with potential participants. For the assessment of scientific and ethical validity, both verbal and written consents were obtained from all the subjects. The study goals, procedures, potential risks, and benefits were further clearly explained directly or indirectly to the volunteers. After being convinced enough by the answers provided by the researcher to their worries, informed consents were then provided either by signing or placing thumb prints on their consent forms. Participants' samples, both blood and stool, were anonymized, while the remaining samples with respect to the hospital biosafety procedures were then destroyed.

### 2.5. Blood and Stool Collection

A fresh bulky stool in a sterile container already labelled with the patient's code was collected and transported to the laboratory as fast as possible (minimum an hour and maximum 2 h) since delay may impede identification (ID) of the causative agents. About 5 mL of blood samples was collected in two tubes, one containing anticoagulant, ethylenediaminetetraacetic acid (EDTA) for biochemical and haematological test and the other without an anticoagulant, for the serological test. Those who were malaria positive were then prescribed drugs by a medical doctor and were given a rendezvous for a period of 2 weeks after which they were again recollected.

#### 2.5.1. Rapid Diagnostic Test (RDT) and Thick Blood Test

RDT (CareStartTM Malaria Pf (HRP2) Ag RDT, Somerset, USA) consisting of a membrane band, precoated with monoclonal antibodies of histidine-rich protein II (HRP-II)–specific mice of *Plasmodium falciparum* was employed. The kit components and samples were initially brought at room temperature before starting. Each cassette was then pulled from its package and put on a flat and dry surface. The test device was labelled with the patient ID. After cleaning the surface of the finger to be sampled with an alcohol-soaked swab, the finger was then pricked with a sterile lancet and the first drop of blood wiped off with cotton wool. The following drop of blood (5 μL) was collected with the aid of a single-use collection loop and then deposited into the round sample well. The diluent solution was then dispensed into the square well of the test device and the results interpreted approximately after 15 min [23, 24].

A thick blood smear consisting of the spreading of a blood drop in a small area of about 1 cm which provides a better opportunity to detect various parasitic forms against a more transparent background was made. A drop of blood was taken and deposited on a previously labelled slide and further spread out using a syringe needle stopper. The blood film was vigorously stirred to release the parasites present in the haematite. After leaving it to dry for at least 30 min, staining of the microscopic slides with the diluted Giemsa solution was done. The slide was then washed with buffered water, rinsed, drained and dried before moving to the microscope for reading using the 100 objective lens and immersion oil.

#### 2.5.2. CD4/CD8 Counts and C-Reactive Protein (CRP) Levels

After recording the patient's data, the EDTA tube was gently shaken to mix the blood sample. The tube was then taken to the probe and the Run button pressed. The flow cytometry was applied to determine the CD4, CD8 T-lymphocyte counts of all the participants using Becton Dickinson's FACS count method (Becton Dickinson, San Jose, CA, USA). CRP Latex (Vitro Scient, Hannover, Germany 2016) was left at room temperature. With the help of a micropipette, a drop (50 μL) of serum obtained from centrifugation was taken and deposited on the circle of the microdilution plate. Two other samples of positive and negative controls were equally realized on the glass slide, and thereafter, 50 μL of reagent latex was deposited beside the drop of serum on the glass slide using the mixing stick, and the serum and CRP latex reagent were uniformly mixed over the entire circle. Immediately, a stopwatch was started, and the slide was gently rocked back and forth for 2 min and then observed for clumping or agglutination macroscopically [25].

#### 2.5.3. Complete Blood Count

Labelled EDTA tubes containing blood were gently agitated to prevent the formation of clots. The sample was then introduced into an automat (Hemascreen 18; Hospitex, Italy) for automatic calculation of red blood cells, white blood cells, platelets, lymphocytes, monocytes, and granulocyte counts after about 9 s.

### 2.6. Sample Collection

Samples of blood and stool were collected under aseptic conditions for 301 patients and were analysed within 1–2 h. After the instant realization of RDT and blood smears for malaria screening, about 10 mL of blood was directly collected into two tubes: one containing the anticoagulant (EDTA) and another tube without an anticoagulant. The blood samples were transported directly for analysis of haematological parameters, CD4 and CD8 T lymphocytes and CRP, while stool samples were sent for microbiological analyses.

### 2.7. Sample Isolation of Enteric Pathogens

Samples were collected from patients in which an initial stool or malaria test has been prescribed by a medical doctor followed by the subsequent processing, making sure stringent measures were respected. For the isolation of pathogenic enteric bacteria, stool suspension was prepared in 5 mL of sterile physiological water. Each suspension was then placed on selective and differential culture media in petri dishes for the isolation of enteric bacteria. The petri dishes together with the specimens were then incubated at 37°C for 24 h.

Colonies of *E. coli* were dark purple with a metallic surface on EMB agar, yellow on Hektoen and their fresh state observation through a microscope showed less mobility. Colonies of *Proteus* spp. were yellow with black centres on Hektoen agar, while on EMB agar, they were greyish marked by the presence of films around with very high mobility. *Klebsiella* spp. colonies were pink with a violet centre, convex without a metallic character on EMB agar, yellowish on Hektoen and not mobile with a fresh state on microscopic observation. The colonies were pale violet, and a metallic character on EMB agar, while it was yellowish on Hektoen and mobile with a fresh state on microscopic observation. *Enterobacter* spp. on EMB agar presented a bluish colour though were yellowish on Hektoen and were mobile upon observation using the microscope. Colonies of *Salmonella* spp. were colourless with a black centre on S-S agar, blue-green with a black centre on Hektoen and greyish transparent on EMB. These colonies were mobile with a fresh state on microscopic observation. The colonies of *Serratia* spp. were colourless with a grey centre on S-S agar and mobile with a fresh state on microscopic observation. Each colony was isolated and purified by culture on nutritive agar at 37°C for 18 h, and confirmation was realized by the study of biochemical profiles on the API 20E gallery, as described by the manufacturer (Bio-Merieux, Lyon, France) [26].

### 2.8. Bacterial Susceptibility Testing to Antibiotics

For the evaluation of susceptibility of the isolates to antibiotics, agar diffusion medium was employed as described by CLSI 2013. A bacterial suspension obtained on agar medium after 24 h of incubation was prepared in the physiological water of turbidity equivalent to the McFarland 0.5 standard and was inoculated on Mueller–Hinton agar that was used as the standard for all the isolates (1.5 × 10^8^ CFU/mL). A suspension of bacteria obtained from nutrient agar after about 18 h of incubation was then prepared using distilled water with an identical turbidity to the reference 0.5 McFarland standard (1.5 × 10^8^ UFC/mL) [27]. Thereafter, antibiotics, precisely 5 disks of antibiotics in one petri dish of 90 mL, were placed on the surface of the agar with the aid of a sterile grip. Each disk was pressed to ensure complete contact with the agar and was distributed such that they were 25 mm apart.

Preincubation at room temperature for 30 min, so as to permit the diffusion of antibiotics, was done followed by complete incubation (Prolabo, Medical international, Rochin, France) at 37°C for 18 h, after which the diameters of the antibiotic inhibition zones were then measured using a graduated ruler and then interpreted as sensitive (S), intermediate (I) or resistant (R) to the antibiotic being tested (Table S1; supporting information) using the break point criteria standards of Clinical and Laboratory Standards Institute (CLSI) [28]. A quality control of antibiotic discs (Oxoid, UK), media (Accumix, Mol, Belgium), and incubation conditions was ensured using *E. coli* ATCC 25922. Isolates showing resistance to three or more categories of antibiotics were considered as multidrug-resistant bacteria [29].

### 2.9. Bacterial Susceptibility Testing of Antimalarial Drugs

The minimal inhibitory concentration (MIC) of each drug was determined by the microdilution method as described earlier [30]. 100 μL of Mueller–Hinton Broth (MHB) was introduced into each well of the 96 microplates.

In the columns of the upper wells, 100 μL of stock solution of antimalarial concentrated at 256 μg/mL was added. The successive serial dilutions of Ratio 2 were carried out followed by the introduction of 100 μL of bacterial inoculum at a density of 2 × 10^8^ CFU/mL into each well for a final volume of 200 μL per well, with extract and antibiotic concentrations varying from 16 μg/mL to 256 μg/mL [31]. The microplates were then sealed and incubated at 37°C for 18–24 h followed by revelation.

After introducing 50 μL of 0.02% para-iodonitrotetrazolium (INT) chloride in each well and reincubating for 30 min, their MICs were then calculated and antimalarial classified.

### 2.10. Statistical Analysis and Data Interpretation

A common database was prepared for all the data followed by statistical analysis using Epi Info Version 7.2.2.6 (CDC, 1600 Clifton Road Atlanta, GA 30329-4027, USA). Significant difference between mono- and coinfected patients was calculated in the case of categorical variables using Chi^2^, followed by a *T*-test for continuous variables. A *p* value of *p* < 0.05 was considered statistically significant.

Antimalarial was classified as very active if MIC < 1 μg/mL, significantly active if 1 < MIC < 10 μg/mL, moderately active if 10 < MIC < 100 μg/mL, low-active if MIC 100 < MIC < 1000 μg/mL and considered not active if MIC > 1000 μg/mL [32], with some specifications depending on the microorganism in question (Table S2; supporting information).

## 3. Results

A total of 301 consented participants in the age group 1–75 years on consultation were recruited, with 127 malaria patients with enteric infections (cases) and 174 nonmalaria patients with enteric infections (control). Out of the 127 malaria patients with enteric infections, 72 (43.31%) were females with an average mean age of 34.24 years, while 55 (56.67%) were males with an average mean age of 31.36 years. Of the 174 nonmalaria patients with enteric infections, 104 (59.09%) were females and 70 (40.23%) were males with average mean ages of 27.00 years and 25.59 years, respectively. The distribution of patients in different age groups shows that there was a significant difference with a *p* value of 0.0014. The highest number of patients with both malaria and enteric infections was obtained in the age group 21–<41: (*N* = 139) with a total of 51 (36.69%) participants followed by the age group 41–<61: (*N* = 61) with a total of 34 (55.74%) participants. The mean age group of the participants was significantly higher in malaria patients with enteric infections (32.99 years) than in nonmalaria patients with enteric infections (26.43 years) with respective ranges of 1–74 and 1–75 (Table 1).

RBCs, WBCs, CD4, CD8, granulocytes, monocytes and platelets were significantly reduced in coinfected patients compared to monoinfected participants with their respective *p* values of 0.0003, 0.0013, 0.00027 < 0.00001, 0.00092, 0.0303 and 0.0491 (Table 2).

**Figure 1:**
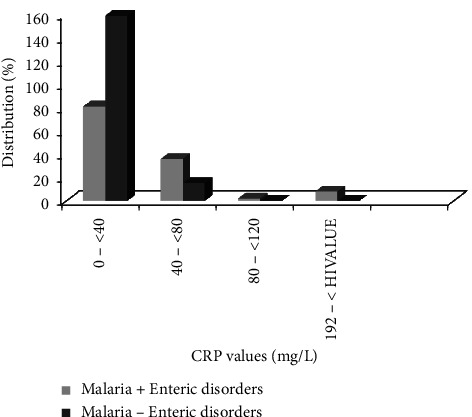
Distribution of CRP in malaria patients with enteric disorders and nonmalaria patients with enteric disorders.

A total of 63.78% of coinfected participants had CRP between 0 < 40 mg/dL against 91.38% of monoinfected individuals. The lowest levels of CRP were between 80 and <120 of 0.00% of coinfected patients and 1.57% of monoinfected patients (Figure 1).

**Figure 2:**
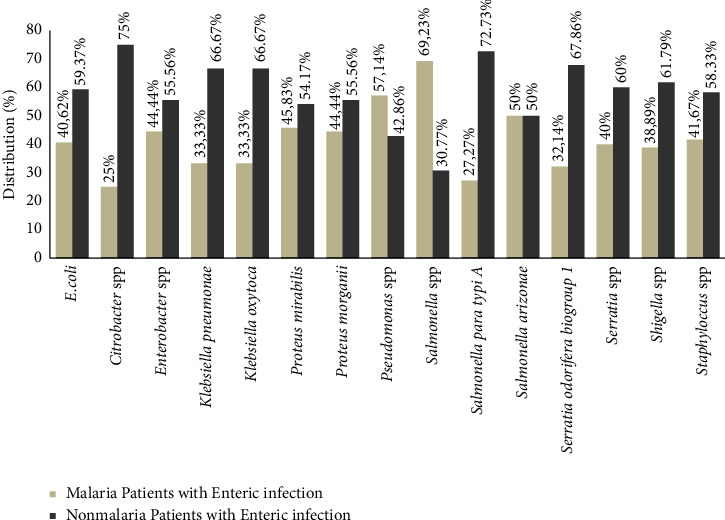
Distribution of bacteria isolates in the population study.

*E. coli* was the pathogenic agent that was frequently isolated in the two populations 26 and 38 for coinfections and monoinfection, respectively (Figure 2).

The most prevalent isolate, *E. coli*, showed high levels of resistance to AMX (57.69%) and CDA (61.54%) in coinfected patients compared to 55.26% and 41.67%, respectively, in monoinfected individuals. The majority of the isolates, such as *Klebsiella* spp., were also highly resistant to CHL (83.33%) and DOX (66.66%) in coinfected individuals compared to 41.67% and 58.33%, respectively, in monoinfected patients. *Enterobacter* spp. showed resistance to CIP (87.50%) and CIP (25.00%) in coinfected and monoinfected participants, respectively (Table 3).

**Figure 3:**
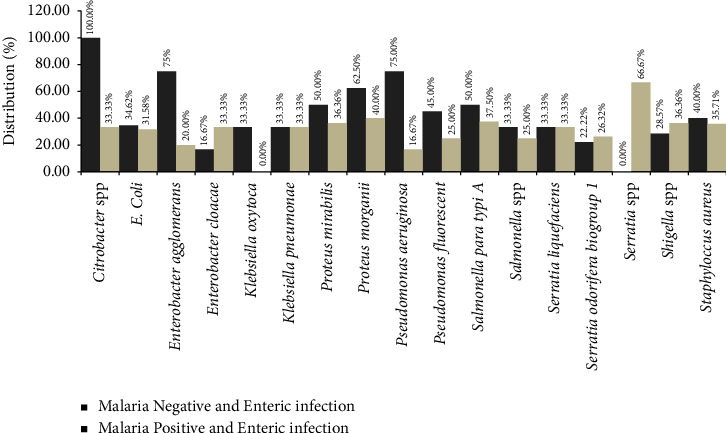
Distribution of multidrug-resistant bacteria isolated from coinfected and monoinfected patients.

Multidrug resistance was equally evaluated and there was evidence that *E. coli* and *Pseudomonas aeruginosa* species showed multidrug resistance of 75.00% each in coinfected patients and 31.58% and 40.00% MDR in monoinfected patients (Figure 3).

Quinine 50.00% and 42.86% presented a MIC of 32 μg/mL on the bacteria isolates from coinfected and monoinfected patients, respectively. Artemether 89 (83.96%), Maloxine 3.94% and Surquina 250 39.37%, MIC of 64 μg/mL, also showed slight activity on bacterial isolates of coinfected and monoinfected patients.

High resistance of *E. coli* was obtained with AKI, AMX and ERY in malaria patients who were under antimalarial drugs (45.93%, 43.75% and 59.37%, respectively) compared to malaria patients who were not under malaria drugs (29.68%, 34.37% and 32.81%, respectively) (Table 4).

In those who were uniquely infected by bacteria, high resistance of *Klebsiella oxytoca*, *E. coli*, *Proteus mirabilis*, and *Enterobacter cloacae* was obtained with CHL, AMX, ERY and COT (55.55%, 57.99%, 69.23% and 61.53%, respectively). Results from their susceptibility test show that *Salmonella* spp. were highly sensitive to CIP, LEV, CAZ and COT (61.53%, 76.92%, 61.53% and 66.66%) among the different groups of participants, that is those having malaria and under treatment, those having malaria and not on treatment and those having only enteric infections (Table 5).

## 4. Discussion

Coinfection is the simultaneous attack of any host by multiple pathogenic species. In the case of this survey, emphasis was laid on malaria (specifically *P. falciparum*) and enteric bacteria coinfections. These are two very highly endemic pathologies common in the tropics, and it is very common nowadays to see patients suffering from the two pathologies.

In this study, 301 participants were recruited, among which 127 were malaria patients with enteric infections (coinfected) and 174 nonmalaria patients with enteric infections (monoinfected). Among the 127 coinfected participants, 72 (43.31%) were females with an average mean age of 34.24 years, while 55 (56.67%) were males with an average mean age of 31.36 years; of the 174 malaria-negative patients with enteric infections, 104 (59.09%) were females and 70 (40.23%) were males with average mean ages of 27.00 years and 25.59 years, respectively. The high population of females obtained in this study could be explained by the fact that women prefer formal hospital treatment than men. This result is in line with the findings of Virginia *et al.*, which revealed an estimated prevalence of 62.00% females and an average mean age of 47.00 years in 2021 [33], stipulating that women usually prefer hospital consultations prior to any medication intake. The highest number of patients with both malaria and enteric infections was obtained in the age group 21–< 41 (*N* = 139) with a total of 51 (36.69%) participants followed by the age group 41–< 61 (*N* = 61) with a total of 34 (55.74%) participants. These results were similar to those obtained by Marbou and Kuete with an estimated (31–40) year of age in 2017 [34]. This might be a result of the fact that the patients included in the study were mostly the active population.

The significant reduction of haematological parameters (RBCs, WBCs, CD4, CD8, granulocytes, monocytes and platelets) in coinfected patients compared to monoinfected participants could be an indication that malaria-induced haemolysis, bone marrow dyserythropoiesis in particular and generalized haematopoietic system disorders have occurred [4, 35]. The decreased RBCs observed in coinfected participants is consistent with the work of Osaro et al. [36], thus attributing anaemia to malaria parasitaemia. Malaria-induced anaemia might certainly result from several factors including the destruction of infected red blood cells which results in decreased RBC counts as well as the rapid removal of both parasitized and nonparasitized red blood cells. Decreased platelets (thrombocytopenia) could be attributed to the sequestration of platelets, splenic pooling of platelets, antibody (IgG)-mediated platelet destruction and adenosine diphosphate (ADP) release following haemolysis of parasitized RBCs, leading to platelet phagocytosis and subsequently decreased number [37].

The overall decrease of CRP level production in coinfected participants compared to monoinfected individuals could be attributed to the fact that marked destruction of CD4 lymphocytes and other cells which serve as producers of pro-inflammatory cytokines had taken place [38, 39].

The predominance of the isolate of *E. coli* identified in the study population is similar to that demonstrated in two regional hospitals in Yaoundé and Banka Ad-Lucem hospital [40, 41]. This could be explained by the fact that *E. coli* is the principal bacterium that normally colonizes the intestine and can become pathogenic in case of immune depression [42]. Data obtained show that the pathogenic *E. coli* genes are widely distributed in different environments of urban wastewater, livestock slaughterhouse wastewater and poultry slaughterhouse as obtained in the work of Afsharnia et al.

The high resistance of *E. coli* to AMX and CDA in coinfected patients compared to monoinfected individuals could be due to the hyperproduction of the ampC beta-lactamase due to the mutation in the ampC promotor/attenuator region and the occurrence of STEC and EPEC virulence genes [43–45].

The resistance of *Klebsiella* spp. could be probable due to the hyperexpression of the efflux pumps of the resistance nodulation cell division (RND) type [46].

The difference in multidrug resistance seen in this study could be due to the effect of the malaria infection that might have been as the result of the host hijack by *Plasmodium falciparum* leading to immunosuppression [47].

Moderate activity on bacterial isolates of coinfected and monoinfected patients by Quinine, Artemether, Maloxine and Surquina 250 with a MIC of 64 μg/mL is a clear indication that the antimalarial compounds Quinine, Artemether, Maloxine and Surquina 250 tested had moderate bacterial activity [32, 48–50]. The MIC of quinine obtained in this study was in line with that obtained by Olateju et al. in 2021, stipulating that quinine is capable of increasing the activity of beta-lactam compounds by increasing their inhibition zones to over 60 mm. Previous studies had also reported antimicrobial activities of 4-aminoquinoline derivatives on both Gram-positive and Gram-negative bacteria complex, though their mechanism of action remains unclear [51]. Other studies by Sallares and Cunnington in 2002, 2005 and 2012 as initially cited have shown that quinine, an antimalarial drug, was able to spontaneously get rid of coinfected individuals of bacterial infections without additional treatment. This might certainly be an indication that antimalarial drugs could be nonspecific and have antibacterial activity.

The high resistance of *E. coli* obtained with AKI, AMX and ERY in malaria patients under malaria drugs compared to malaria patients who were not under malaria drugs could be due to the increase resistance, as the result of partial action of the antimalarial drugs that activated the efflux pumps, thus increasing the ability of the bacteria to expel drugs out of their cells, thus increasing bacterial resistance. This is a clear indication that antimalarial treatment might certainly have a clear influence on microbial resistance.

The resistance of *Klebsiella oxytoca*, *E. coli, Proteus mirabilis and Enterobacter cloacae* to CHL, AMX, ERY and COT (55.55%, 57.99%, 69.23% and 61.53%, respectively) obtained in the course of this work was also demonstrated in the previous studies of Ornela *et al.* at the Banka Ad-Lucem Hospital in 2019 [50]. This could be due to the overexpression of efflux pumps of the RND by isolates of *Klebsiella* capable of constantly ejecting the antibiotics from the bacteria cell, the action of some key enzymes capable of destroying the beta-lactam compounds (action of beta-lactamases), making antibiotics inefficient [52]. Moreover, antimicrobial resistance patterns could certainly be influenced by the source of the isolates, classes of antimicrobial agents, pressure exerted by antimicrobial use and geographic location.

Susceptibility tests revealed that most isolates of *Salmonella* spp. from patients that had only related enteric infections were susceptible to CIP, LEV, CAZ and COT. These results imply that the antibiotics in question could be used for empirical treatment of these enteric pathogens in areas where microbial culture facilities are problematic as revealed by the works of Getie et al. in northwest Ethiopia [53].

Nonetheless, from this study, the emergence of resistance obtained might certainly be as the result of the frequent intake of antimalarials and antibiotics in the treatment of plasmodium and bacterial infections, respectively. One of the most plausible mechanisms that might explain this phenomenon might be the induction of nonspecific efflux pump by any toxic substance present in the organism.

This study was limited to a certain extent due to the fact that some of the participants who were administered antimalarial and given a rendezvous for follow-up could not present characteristics permitting them to be included once more in the study, so a greater percentage of the sample size was obtained barely on the past history of the patients.

## 5. Conclusion

This study clearly demonstrated the association of RBCs, WBCs, CD4, CD8, granulocytes, monocytes and platelets, and resistant profile of enteric bacteria from patients who were malaria-positive and having bacterial infections compared to those who were malaria-negative and having enteric infections on consultation at the Dschang Regional Hospital. Significant decrease in these haematological parameters was observed in coinfected patients compared to monoinfected participants. No significant association was observed in HGB, LYM, HCT and serum CRP of coinfected patients with respect to monoinfected patients. High levels of resistance were observed with AMX and CDA, with an overall increase of resistance in coinfected patients compared to monoinfected participants. Antimalarial drugs such as quinine, artemether, G-Cospe, Artesunate/amodiaquine, Maloxine, Nemether and Surquina 250 showed moderate antibiotic activity. With respect to the influence of malaria treatment on bacterial resistance, it was seen that resistance increased in most cases proportionately with malaria treatment. Intake of antimalarial drugs might certainly have an influence on the emergence of bacteria resistance in the case of malaria bacterial coinfection, and therefore, adequate management and effective control programmes are necessary to reduce morbidity and mortality.

## Figures and Tables

**Table 1:** Sociodemographic characteristics of the population.

Parameters		Malaria-positive and enteric infection (*N* = 127)	Nonmalaria with enteric infection (*N* = 174)	*p* value
Sex	Female	72 (56.67%)	104 (59.09%)	0.5922
	Male	55 (43.31%)	70 (40.23%)	

Age groups (years)	1–<21: (*N* = 88)	30 (34.09%)	58 (65.91%)	**0.0014**
	21–<41: (*N* = 139)	51 (36.69%)	88 (63.31%)	
	41–<61: (*N* = 61)	34 (55.74%)	27 (44.26%)	
	61–<75: (*N* = 12)	12 (100%)	0 (0.00%)	
	75–<Hi: (*N* = 1)	0 (0%)	1 (100%)	

Mean age; mean ± SD [min–max]	Total	32.99 ± 19.21[1–74]	26.43 ± 15.37[1–75]	**0.0011**
	Female	34.24 ± 17.05[7–74]	27 ± 14.81[1–75]	**0.0048**
	Male	31.36 ± 21.78[1–65]	25.59 ± 16.23[1–59]	**< 0.0001**

*Note:* Bold values indicate significant differences.

**Table 2:** Distribution of haematological parameters in the two populations (malarial patients with enteric disorders and nonmalaria patients with enteric disorders).

Parameters	Malaria patients with enteric disorders (*n* = 127)		Nonmalaria with enteric disorders (*n* = 174)		*p* value
	Mean value ± SD range (Min-Max)		Mean value ± SD range (Min-Max)		
Red blood cell (×10^6^ µL)	1.45 ± 4.92	1.45–7.48	4.77 ± 4.15	1.45–8.25	0.0003
White blood cells (×10^3^ µL)	7.23–9.6	2.60–9.9	11.1 ± 4.3	6.60 ± 4.5	0.0013
Platelets (×10^3^ µL)	187.67 ± 81.91	60–440	221.24 ± 75.14	60–440	0.00027
Granulocytes (%)	74.2 ± 58.5	19.8–91.3	79.2 ± 73.4	28.5–92.6	0.0303
Monocytes (%)	4.0 ± 2.5	1.4–6.4	4.04.4 ± 2.4	1.4–7.3	0.0491
Haemoglobin g (Dl)	14.1 ± 13.1	10.0–17.4	14.5 ± 13.1	10.1–13.1	0.1735
Lymphocytes (%)	38.1 ± 36.1	23.4–38.7	38.5 ± 44.8	26.1–49.6	0.1070
Haematocrit (%)	40.0 ± 34.4	30.8–40.0	40.7 ± 38.3	34.6–40.7	0.0056
CD4 cells (mm^3^)	471.06 ± 256.28	200.00–1420.00	600.00 ± 154.57	208.05–1000	< 0.00001
CD8 cells (mm^3^)	247.26 ± 107.10	120.00–659.00	287.10 ± 97.36	108.00–659.00	0.00092

**Table 3:** Distribution of susceptibility profile of bacteria with respect to the populations under study.

**ATB**	**Profile**	***C.* spp**		***E. coli***		***E. agglomerans***		***E. cloacae***		***K. oxytoca***		***K. pneumoniae***	
		**M + Ent**	**M − Ent**	**M + Ent**	**M − Ent**	**M + Ent**	**M − Ent**	**M + Ent**	**M − Ent**	**M + Ent**	**M − Ent**	**M + Ent**	**M − Ent**

AMX	I	0 (0.00%)	0 (0.00%)	1 (3.85%)	3 (7.89%)	0 (0.00%)	0 (0.00%)	0 (0.00%)	0 (0.00%)	0 (0.00%)	0 (0.00%)	1 (16.67%)	0 (0.00%)
	R	1 (100.00%)	1 (33.33%)	15 (57.69%)	21 (55.26%)	1 (25.00%)	7 (70.00%)	2 (25.00%)	4 (80.00%)	2 (66.67%)	1 (16.67%)	2 (33.33%)	5 (41.67)
	S	0 (0.00%)	2 (66.67%)	10 (38.46%)	14 (36.84%)	3 (75.00%)	3 (30.00%)	6 (75.00%)	1 (20.00%)	1 (33.33%)	5 (83.33%)	3 (50.00%)	7 (58.33%)

CAZ	I	0 (0.00%)	0 (0.00%)	0 (0.00%)	0 (0.00%)	0 (0.00%)	0 (0.00%)	1 (12.50%)	0 (0.00%)	0 (0.00%)	0 (0.00%)	0 (0.00%)	1 (8.33%)
	R	0 (0.00%)	2 (66.67%)	14 (53.85%)	20 (52.63%)	2 (50.00%)	4 (40.00%)	2 (25.00)	3 (40.00%)	1 (33.33%)	4 (66.67%)	6 (100.00%)	5 (41.67%)
	S	1 (100.00%)	1 (33.33%)	12 (46.15%)	18 (47.37%)	2 (50.00%)	6 (60.00%)	5 (62.50%)	2 (60.00%)	2 (66.67%)	2 (33.33%)	0 (0.00%)	6 (50.00%)

CIP	I	0 (0.00%)	0 (0.00%)	0 (0.00%)	0 (0.00%)	0 (0.00%)	0 (0.00%)	1 (12.50%)	0 (0.00%)	0 (0.00%)	0 (0.00%)	0 (0.00%)	0 (0.00%)
	R	0 (0.00%)	0 (0.00%)	12 (46.15%)	19 (50.00%)	1 (25.00%)	4 (40.00%)	5 (62.50%)	1 (25.00%)	2 (66.67%)	1 (25.00%)	2 (33.33%)	9 (75.00%)
	S	1 (100.00%)	3 (100.00%)	13 (53.85%)	19 (50.00%)	3 (75.00%)	6 (60.00%)	2 (25.00%)	3 (75.00%)	1 (33.33%)	3 (75.00%)	4 (66.67%)	3 (25.00%)

COT	I	0 (0.00%)	0 (0.00%)	1 (3.85%)	3 (7.89%)	0 (0.00%)	1 (10.00%)	0 (0.00%)	1 (11.11%)	0 (0.00%)	0 (0.00%)	0 (0.00%)	0 (0.00%)
	R	0 (0.00%)	1 (33.33%)	11 (42.31%)	21 (55.26%)	1 (25.00%)	6 (60.00%)	5 (83.33%)	4 (44.44%)	0 (0.00%)	4 (66.67%)	3 (50.00%)	9 (75.00%)
	S	1 (100.00%)	2 (66.67%)	14 (53.85%)	14 (36.84%)	3 (75.00%)	3 (30.00%)	3 (16.67%)	4 (44.44%)	3 (100.00%)	2 (33.33%)	3 (50.00%)	3 (25.00%)

CDA	I	0 (0.00%)	1 (33.33%)	1 (3.85%)	1 (2.63%)	0 (0.00%)	0 (0.00%)	0 (0.00%)	0 (0.00%)	0 (0.00%)	0 (0.00%)	0 (0.00%)	0 (0.00%)
	R	1 (100.00%)	2 (66.67%)	16 (61.54%)	22 (57.89%)	2 (50.00%)	4 (40.00%)	5 (62.50%)	2 (40.00%)	2 (66.67%)	3 (50.00%)	4 (66.67%)	8 (66.67%)
	S	0 (0.00%)	0 (0.00%)	9 (34.62%)	15 (39.47%)	2 (50.00%)	6 (60.00%)	3 (37.50%)	3 (60.00%)	1 (33.33%)	3 (50.00%)	2 (33.33%)	4 (33.33%)

LEV	I	0 (0.00%)	1 (33.33%)	3 (11.54%)	5 (13.16%)	0 (0.00%)	0 (0.00%)	1 (11.11%)	2 (40.00%)	0 (0.00%)	1 (16.67%)	0 (0.00%)	0 (0.00%)
	R	1 (100.00%)	1 (33.33%)	13 (50.00%)	16 (42.11%)	1 (25.00%)	5 (50.00%)	5 (55.55%)	3 (60.00%)	3 (100.00%)	2 (33.33%)	4 (66.67%)	8 (66.67%)
	S	0 (0.00%)	1 (33.33%)	10 (38.46%)	17 (44.74%)	3 (75.00%)	5 (50.00%)	3 (33.33%)	0 (0.00%)	0 (0.00%)	3 (50.00%)	2 (33.33%)	4 (33.33%)

DOX	I	0 (0.00%)	0 (0.00%)	3 (11.54%)	1 (2.63%)	1 (25.00%)	0 (0.00%)	0 (0.00%)	0 (0.00%)	0 (0.00%)	1 (16.67%)	0 (0.00%)	0 (0.00%)
	R	1 (100.00%)	3 (100.00%)	14 (53.85%)	23 (60.53%)	1 (25.00%)	7 (70.00%)	5 (62.50%)	4 (80.00%)	2 (66.67%)	4 (66.67%)	5 (83.33%)	7 (58.33%)
	S	0 (0.00%)	0 (0.00%)	9 (34.62)	14 (36.84%)	2 (50.00%)	3 (30.00%)	3 (37.50%)	1 (20.00%)	1 (33.33%)	1 (16.67%)	1 (16.67%)	5 (41.67%)

CHL	I	0 (0.00%)	0 (0.00%)	1 (3.85%)	2 (5.26%)	0 (0.00%)	1 (10.00%)	0 (0.00%)	0 (0.00%)	0 (0.00%)	0 (0.00%)	0 (0.00%)	3 (25.00%)
	R	0 (0.00%)	1 (33.33%)	15 (57.59%)	20 (52.63%)	2 (50.00%)	5 (50.00%)	4 (50.00%)	3 (60.00%)	1 (33.33%)	4 (66.67%)	3 (50.00%)	5 (41.67%)
	S	1 (100.00%)	2 (66.67%)	10 (38.46%)	16 (42.11%)	2 (50.00%)	4 (40.00%)	4 (50.00%)	2 (40.00%)	2 (66.67%)	2 (33.33%)	3 (50.00%)	4 (33.33%)

ERY	I	0 (0.00%)	1 (33.33%)	2 (7.69%)	2 (5.26%)	0 (0.00%)	0 (0.00%)	1 (12.50%)	0 (0.00%)	0 (0.00%)	0 (0.00%)	0 (0.00%)	1 (8.33%)
	R	0 (0.00%)	1 (33.33%)	14 (53.85%)	16 (42.11%)	2 (50.00%)	8 (80.00%)	3 (37.50%)	3 (60.50%)	3 (100.00%)	5 (83.33%)	3 (50.00%)	9 (75.00%)
	S	1 (100.00%)	1 (33.33%)	10 (38.45%)	20 (52.23%)	2 (50.00%)	2 (20.00%)	4 (50.00%)	4 (80.00%)	0 (0.00%)	1 (16.67%)	3 (50.00%)	2 (16.67%)

SXT	I	0 (0.00%)	1 (33.33%)	3 (11.54%)	1 (2.63%)	0 (0.00%)	0 (0.00%)	0 (0.00%)	0 (0.00%)	0 (0.00%)	0 (0.00%)	0 (0.00%)	0 (0.00%)
	R	1 (100.00%)	1 (33.33%)	14 (53.85%)	23 (60.53%)	3 (75.00%)	4 (40.00%)	5 (62.50%)	3 (60.00%)	2 (66.67%)	4 (66.67%)	2 (33.33%)	5 (41.67%)
	S	0 (0.00%)	1 (33.33%)	9 (34.62)	14 (36.84%)	1 (25.00%)	6 (60.00%)	3 (37.50%)	2 (40.00%)	1 (33.33%)	2 (33.33%)	4 (66.67%)	7 (58.33%)

AMK	I	0 (0.00%)	0 (0.00%)	1 (3.85%)	2 (5.26%)	0 (0.00%)	1 (10.00%)	1 (12.50%)	1 (20.00%)	0 (0.00%)	0 (0.00%)	0 (0.00%)	0 (0.00%)
	R	0 (0.00%)	1 (33.33%)	13 (50.00%)	21 (55.26%)	2 (50.00%)	4 (40.00%)	4 (50.00%)	3 (60.00%)	2 (66.67%)	4 (66.67%)	4 (66.67%)	6 (50.00%)
	S	1 (100.00%)	2 (66.67%)	12 (46.15%)	15 (39.47%)	2 (50.00%)	5 (50.00%)	3 (37.50%)	1 (20.00%)	1 (33.33%)	2 (33.33%)	2 (33.33%)	6 (50.00%)

AMC	I	0 (0.00%)	1 (33.33%)	0 (0.00%)	2 (5.26%)	0 (0.00%)	2 (20.00%)	0 (0.00%)	0 (0.00%)	0 (0.00%)	0 (0.00%)	1 (16.67%)	0 (0.00%)
	R	0 (0.00%)	2 (66.67%)	16 (61.54%)	17 (44.74%)	2 (50.00%)	5 (50.00%)	7 (87.50%)	2 (40.00%)	2 (66.67%)	5 (83.33%)	3 (50.00%)	8 (66.67%)
	S	1 (100.00%)	0 (0.00%)	10 (38.46%)	19 (50.00%)	2 (50.00%)	3 (30.00%)	1 (12.50%)	3 (60.00%)	1 (33.33%)	1 (16.67%)	2 (33.33%)	4 (33.33%)

**ATB**	**Profile**	***P. mirabilis***		***P. morganii***		***Ps. aeruginosa***		***Ps. fluorescent***		***S. paratyphi A***		***S. arizonae***	
		**M + Ent**	**M − Ent**	**M + Ent**	**M − Ent**	**M + Ent**	**M − Ent**	**M + Ent**	**M − Ent**	**M + Ent**	**M − Ent**	**M + Ent**	**M − Ent**

AMX	I	0 (0.00%)	0 (0.00%)	0 (0.00%)	0 (0.00%)	2 (25.00%)	1 (13.5)	0 (0.00%)	0 (0.00%)	0 (0.00%)	1 (12.50)	0 (0.00%)	0 (0.00%)
	R	5 (45.45%)	10 (72.73%)	3 (37.50%)	5 (50.00%)	5 (62.50%)	3 (37.5%)	2 (50.00%)	0 (0.00%)	1 (100.00%)	3 (37.50%)	0 (0.00%)	3 (50.00%)
	S	6 (54.54%)	3 (27.27)	5 (62.50%)	5 (50.00%)	1 (12.50%)	4 (50.00%)	2 (50.00%)	1 (100.00%)	0 (0.00%)	4 (50.00%)	6 (100.00%)	3 (50.00%)

CAZ	I	1 (9.09%)	0 (0.00%)	0 (0.00%)	0 (0.00%)	0 (0.00%)	1 (12.50%)	0 (0.00%)	0 (0.00%)	0 (0.00%)	0 (0.00%)	1 (16.67%)	1 (16.67%)
	R	4 (36.36%)	6 (46.15%)	3 (37.50%)	6 (60.00%)	5 (62.50%)	5 (62.50%)	3 (75.00%)	1 (100.00%)	3 (100.00%)	2 (25.00%)	3 (50.00%)	3 (50.00%)
	S	6 (54.54%)	7 (53.84%)	5 (62.50%)	4 (40.00%)	3 (37.50%)	2 (25.00%)	1 (25.00%)	0 (0.00%)	0 (0.00%)	6 (75.00%)	2 (33.33%)	2 (33.33%)

CIP	I	0 (0.00%)	0 (0.00%)	0 (0.00%)	0 (0.00%)	0 (0.00%)	0 (0.00%)	0 (0.00%)	0 (0.00%)	0 (0.00%)	0 (0.00%)	0 (0.00%)	0 (0.00%)
	R	5 (50.00%)	1 (50.00%)	6 (75.00%)	7 (70.00%)	5 (83.33%)	3 (50.00%)	3 (75.00%)	1 (100.00%)	2 (66.66%)	4 (50.00%)	4 (66.67%)	4 (66.67%)
	S	5 (50.00%)	1 (50.00%)	2 (25.00%)	3 (30.00%)	1 (16.66%)	3 (50.00%)	1 (25.00%)	0 (0.00%)	1 (33.33%)	4 (50.00%)	2 (33.33%)	2 (33.33%)

COT	I	0 (0.00%)	0 (0.00%)	1 (12.50%)	0 (0.00%)	0 (0.00%)	2 (25.00%)	0 (0.00%)	0 (0.00%)	0 (0.00%)	1 (12.50%)	0 (0.00%)	1 (16.67%)
	R	5 (50.00%)	7 (63.64%)	5 (62.50%)	7 (70.00%)	4 (57.14%)	5 (62.50%)	2 (50.00%)	1 (100.00%)	2 (66.66%)	3 (37.50%)	3 (60.00%)	5 (83.33%)
	S	5 (50.00%)	4 (36.36%)	2 (25.00%)	3 (30.00%)	3 (42.85%)	1 (12.50%)	2 (50.00%)	0 (0.00%)	1 (33.33%)	4 (50.00%)	2 (40.00%)	0 (0.00%)

CDA	I	0 (0.00%)	0 (0.00%)	0 (0.00%)	0 (0.00%)	0 (0.00%)	0 (0.00%)	0 (0.00%)	0 (0.00%)	0 (0.00%)	0 (0.00%)	1 (16.67%)	0 (0.00%)
	R	6 (54.54%)	7 (63.64%)	5 (62.50%)	5 (50.00%)	5 (62.50%)	2 (25.00%)	3 (75.00%)	1 (100.00%)	2 (66.67%)	4 (50.00%)	4 (66.67%)	2 (33.33%)
	S	5 (45.45%)	6 (36.36%)	3 (37.50%)	5 (50.00%)	3 (37.50)	6 (75.00%)	1 (25.00%)	0 (0.00%)	1 (33.33%)	4 (50.00%)	1 (16.67%)	4 (66.67%)

LEV	I	1 (9.09%)	1 (7.69%)	0 (0.00%)	0 (0.00%)	1 (12.50%)	1 (12.50%)	0 (0.00%)	0 (0.00%)	0 (0.00%)	0 (0.00%)	1 (16.67%)	1 (16.67%)
	R	5 (45.45%)	8 (61.53%)	7 (87.50%)	7 (70.00%)	4 (50.00%)	5 (62.50%)	3 (75.00%)	1 (100.00%)	1 (33.33%)	1 (12.50%)	2 (33.33%)	5 (83.33%)
	S	5 (45.45%)	4 (30.76%)	1 (12.50%)	3 (30.00%)	3 (37.50)	2 (25.00)	1 (25.00%)	0 (0.00%)	2 (67.67%)	7 (87.50%)	3 (50.00%)	0 (0.00%)

DOX	I	0 (0.00%)	0 (0.00%)	0 (0.00%)	0 (0.00%)	0 (0.00%)	0 (0.00%)	1 (25.00%)	0 (0.00%)	0 (0.00%)	1 (12.50%)	0 (0.00%)	0 (0.00%)
	R	5 (45.45%)	7 (53.84)	5 (62.50%)	7 (70.00%)	5 (62.50%)	6 (85.72%)	1 (25.00%)	1 (100.00%)	3 (100.00%)	3 (37.50%)	5 (83.33%)	4 (66.67%)
	S	6 (54.54%)	6 (46.15)	3 (37.50%)	3 (30.00%)	3 (37.50%)	1 (14.28%)	2 (50.00%)	0 (0.00%)	0 (0.00%)	4 (50.00%)	1 (16.67%)	2 (33.33%)

CHL	I	1 (9.09%)	1 (7.69)	0 (0.00%)	0 (0.00%)	0 (0.00%)	1 (12.50%)	0 (0.00%)	0 (0.00%)	2 (66.66%)	0 (0.00%)	0 (0.00%)	2 (33.33%)
	R	3 (27.27%)	7 (53.84)	5 (62.50%)	6 (60.00%)	7 (87.50%)	6 (75.00%)	2 (50.50%)	0 (0.00%)	1 (33.33%)	8 (100.00%)	7 (77.77%)	3 (50.00%)
	S	7 (63.63%)	5 (38.46%)	3 (37.50%)	4 (40.00%)	1 (12.50%)	1 (12.50%)	2 (50.50%)	1 (100.00%)		0 (0.00%)	2 (22.22%)	1 (16.67%)

ERY	I	1 (9.09%)	2 (15.38%)	0 (0.00%)	0 (0.00%)	0 (0.00%)	1 (12.50%)	0 (0.00%)	0 (0.00%)	0 (0.00%)	0 (0.00%)	0 (0.00%)	0 (0.00%)
	R	4 (36.36%)	7 (53.84%)	4 (50.00%)	7 (70.00%)	5 (62.50%)	1 (12.50%)	4 (100.00%)	0 (0.00%)	3 (100.00%)	3 (37.50%)	4 (66.67%)	5 (83.33%)
	S	6 (54.54%)	4 (30.76%)	4 (50.00%)	3 (30.00%)	3 (37.50%)	6 (75.00%)	0 (0.00%)	1 (100.00%)	0 (0.00%)	5 (62.50%)	2 (33.33%)	1 (16.67%)

SXT	I	0 (0.00%)	0 (0.00%)	0 (0.00%)	0 (0.00%)	0 (0.00%)	0 (0.00%)	0 (0.00%)	0 (0.00%)	0 (0.00%)	0 (0.00%)	0 (0.00%)	0 (0.00%)
	R	8 (72.72%)	8 (61.53%)	4 (50.00%)	6 (60.00%)	6 (75.00%)	4 (57.14%)	2 (50.00%)	1 (100.00%)	3 (100.00%)	3 (37.50%)	4 (66.67%)	5 (83.33%)
	S	3 (27.27%)	5 (38.46%)	4 (50.00%)	4 (40.00%)	2 (25.00%)	3 (42.85%)	2 (50.00%)	0 (0.00%)	0 (0.00%)	5 (62.50%)	2 (33.33%)	1 (16.67%)

AMK	I	1 (9.09%)	0 (0.00%)	0 (0.00%)	1 (10.00%)	0 (0.00%)	0 (0.00%)	0 (0.00%)	0 (0.00%)	0 (0.00%)	0 (0.00%)	0 (0.00%)	0 (0.00%)
	R	4 (36.36%)	10 (76.92%)	6 (75.00%)	3 (30.00%)	7 (87.50%)	5 (62.50%)	4 (100.00%)	1 (100.00%)	2 (66.66%)	5 (62.50%)	4 (66.67%)	5 (83.33%)
	S	6 (54.54%)	3 (23.07%)	2 (25.00%)	6 (60.00%)	1 (12.50%)	3 (37.50%)	0 (0.00%)	0 (0.00%)	1 (33.33%)	3 (37.50%)	2 (33.33%)	1 (16.67%)

AMC	I	1 (9.09%)	1 (7.69%)	0 (0.00%)	1 (10/00%)	0 (0.00%)	1 (14.28%)	0 (0.00%)	0 (0.00%)	0 (0.00%)	0 (0.00%)	1 (16.67%)	0 (0.00%)
	R	5 (54.54%)	7 (53.84%)	5 (62.50%)	3 (30.00%)	4 (50.00%)	4 (57.14%)	1 (25.00%)	1 (100.00%)	1 (33.33%)	5 (62.50%)	5 (83.33%)	2 (33.33%)
	S	5 (54.54%)	5 (38.46%)	3 (37.50%)	6 (60.00%)	4 (50.00%)	2 (28.57%)	3 (75.00%)	0 (0.00%)	2 (66.66%)	3 (37.50%)	0 (0.00%)	4 (66.67%)

**ATB**	**Profile**	***S.* spp**		***Ser. liquefaciens***		***Ser o biogroup 1***		***Shigella* spp**		***Staph aureus***			
		**M + Ent**	**M − Ent**	**M + Ent**	**M − Ent**	**M + Ent**	**M − Ent**	**M + Ent**	**M − Ent**	**M + Ent**	**M − Ent**		

AMX	I	0 (0.00%)	1 (25.00%)	1 (33.33%)	0 (0.00%)	0 (0.00%)	0 (0.00%)	1 (14.29%)	1 (9.09%)	0 (0.00%)	0 (0.00%)		
	R	8 (88/89%)	2 (50.00%)	1 (33.33%)	1 (33.33%)	2 (22.22%)	12 (63.16%)	3 (42.86%)	6 (54.55%)	4 (40.00%)	7 (50.00%)		
	S	1 (11.11%)	1 (25.00%)	1 (33.33%)	2 (66.67%)	7 (77.78%)	7 (36.85)	3 (42.86%)	4 (36.36%)	6 (60.00%)	7 (50.00%)		

CAZ	I	0 (0.00%)	0 (0.00%)	0 (0.00%)	0 (0.00%)	0 (0.00%)	0 (0.00%)	0 (0.00%)	0 (0.00%)	0 (0.00%)	0 (0.00%)		
	R	7 (77.78%)	3 (75.00%)	2 (66.67%)	3 (100.00%)	6 (66.67%)	14 (73.68%)	4 (57.14%)	8 (72.73%)	7 (70.00%)	9 (64.29%)		
	S	2 (22.22%)	1 (25.00%)	1 (33.33%)	0 (0.00%)	3 (33.33%)	5 (26.32%)	3 (42.86%)	1 (33.33%)	3 (30.00%)	5 (35.71%)		

CIP	I	0 (0.00%)	0 (0.00%)	0 (0.00%)	0 (0.00%)	0 (0.00%)	0 (0.00%)	0 (0.00%)	0 (0.00%)	1 (10.00%)	1 (7.14%)		
	R	8 (88.89%)	3 (75.00%)	2 (66.67%)	1 (33.33%)	7 (77.78%)	8 (44.44%)	5 (71.43%)	4 (36.36%)	4 (40.00%)	7 (50.00%)		
	S	1 (11.11%)	1 (25.00%)	1 (33.33%)	2 (66.67%)	2 (22.22%)	10 (55.56%)	2 (28.57%)	7 (63.64%)	5 (50.00%)	6 (42.86%)		

COT	I	1 (11.11%)	0 (0.00%)	0 (0.00%)	0 (0.00%)	0 (0.00%)	2 (10.53%)	0 (0.00%)	0 (0.00%)	2 (20.00%)	1 (7.14%)		
	R	7 (77.78%)	3 (75.00%)	3 (100.00%)	0 (0.00%)	5 (55.56%)	10 (52.63%)	4 (57.14%)	6 (54.55%)	5 (50.00%)	5 (35.71%)		
	S	1 (11.11%)	1 (25.00%)	0 (0.00%)	1 (100.00%)	4 (44.44)	7 (36.84%)	3 (42.86%)	5 (45.45%)	3 (30.00%)	8 (57.14%)		

CDA	I	0 (0.00%)	0 (0.00%)	0 (0.00%)	0 (0.00%)	0 (0.00%)	0 (0.00%)	0 (0.00%)	0 (0.00%)	0 (0.00%)	0 (0.00%)		
	R	5 (71.43%)	3 (75.00%)	1 (33.33%)	0 (0.00%)	2 (22.22%)	6 (31.58%)	5 (71.43%)	7 (63.64%)	8 (80.00%)	8 (57.14%)		
	S	2 (28.57%)	1 (25.00%)	2 (66.67%)	3 (100.00%)	7 (77.78%)	13 (68.42%)	2 (28.57%)	4 (36.36%)	2 (20.00%)	6 (42.86%)		

LEV	I	1 (11.11%)	1 (25.00%)	1 (33.33%)	0 (0.00%)	0 (0.00%)	1 (5.26%)	0 (0.00%)	0 (0.00%)	0 (0.00%)	1 (7.14%)		
	R	5 (55.56%)	0 (0.00%)	2 (66.67%)	0 (0.00%)	5 (55.56%)	6 (31.58%)	4 (57.14%)	4 (36.36%)	8 (80.00%)	8 (57.14%)		
	S	3 (33.33%)	3 (75.00%)	0 (0.00%)	3 (100.00%)	4 (44.44%)	12 (63.16%)	3 (42.86%)	7 (63.64%)	2 (20.00%)	5 (35.71%)		

DOX	I	1 (11.11%)	0 (0.00%)	0 (0.00%)	0 (0.00%)	0 (0.00%)	1 (5.26%)	0 (0.00%)	0 (0.00%)	0 (0.00%)	0 (0.00%)		
	R	7 (77.78%)	2 (50.00%)	2 (66.67%)	6 (66.67%)	6 (66.67)	12 (63.16%)	4 (57.14%)	7 (63.64%)	9 (90.00%)	9 (64.29%)		
	S	1 (11.11%)	2 (50.00%)	1 (33.33%)	1 (33.33%)	2 (33.33%)	1 (33.33%)	3 (42.86%)	4 (36.36%)	1 (10.00%)	5 (35.71%)		

CHL	I	0 (0.00%)	1 (25.00%)	0 (0.00%)	0 (0.00%)	0 (0.00%)	2 (10.53%%)	0 (0.00%)	0 (0.00%)	0 (0.00%)	0 (0.00%)		
	R	7 (77.77%)	1 (25.00%)	1 (33.33%)	0 (0.00%)	4 (44.44%)	7 (36.84%)	4 (57.14%)	5 (45.45%)	6 (60.00%)	5 (35.71%)		
	S	2 (22.22%)	2 (50.00%)	2 (66.67%)	3 (100.00%)	5 (55.56%)	10 (52.63%)	3 (42.86%)	6 (54.55%)	4 (40.00%)	9 (64.29%)		

PEN-G	I	NT	NT	NT	NT	NT	NT	NT	NT	0 (0.00%)	1 (8.33%)		
	R	NT	NT	NT	NT	NT	NT	NT	NT	9 (90.00%)	6 (50.00%)		
	S	NT	NT	NT	NT	NT	NT	NT	NT	1 (10.00%)	5 (41.67%)		

ERY	I	0 (0.00%)	0 (0.00%)	0 (0.00%)	0 (0.00%)	0 (0.00%)	1 (5.26%)	0 (0.00%)	0 (0.00%)	0 (0.00%)	2 (14.29%)		
	R	7 (77.78%)	1 (25.00%)	2 (66.67%)	2 (66.67%)	5 (55.56%)	8 (42.11%)	4 (57.14%)	6 (54.55%)	10 (100.00%)	10 (71.43%)		
	S	2 (22.22%)	3 (75.00%)	1 (33.33%)	1 (33.33%)	4 (44.44%)	10 (52.63%)	3 (42.86%)	5 (45.45%)	0 (0.00%)	2 (14.29%)		

SXT	I	0 (0.00%)	0 (0.00%)	1 (33.33%)	0 (0.00%)	0 (0.00%)	1 (5.26%)	0 (0.00%)	0 (0.00%)	0 (0.00%)	0 (0.00%)		
	R	6 (66.67%)	3 (75.00%)	0 (0.00%)	3 (100.00%)	4 (44.44%)	10 (52.63%)	5 (71.43%)	4 (36.36%)	8 (80.00%)	7 (50.00%)		
	S	3 (33.33%)	1 (25.00%)	2 (66.67%)	0 (0.00%)	5 (55.55%)	8 (42.11%)	2 (28.57%)	7 (63.64%)	2 (20.00%)	7 (50.00%)		

AMK	I	0 (0.00%)	0 (0.00%)	0 (0.00%)	0 (0.00%)	0 (0.00%)	0 (0.00%)	0 (0.00%)	2 (18.18%)	0 (0.00%)	1 (7.14%)		
	R	8 (88.89%)	3 (75.00%)	2 (66.67%)	3 (100/00%)	9 (100.00%)	15 (78.95%)	4 (57.14%)	5 (45.45%)	7 (70.00%)	8 (57.14%)		
	S	1 (11.11%)	1 (25.00%)	1 (33.33%)	0 (0.00%)	0 (0.00%)	4 (21.05%)	3 (42.86%)	4 (36.36%)	3 (30.00%)	5 (35.71%)		

AMC	I	0 (0.00%)	0 (0.00%)	0 (0.00%)	0 (0.00%)	0 (0.00%)	1 (5.26%)	0 (0.00%)	0 (0.00%)	0 (0.00%)	0 (0.00%)		
	R	6 (66.67%)	3 (75.00%)	2 (66.67%)	2 (66.67%)	8 (88.89%)	14 (73.68)	4 (57.14%)	6 (54.55%)	8 (80.00%)	9 (64.29%)		
	S	3 (33.33%)	1 (25.00%)	1 (33.33%)	1 (33.33%)	1 (11.11%)	4 (21.05%)	3 (42.86%)	5 (45.45%)	1 (20.00%)	5 (35.71%)		

Abbreviations: AMC, amoxicillin + clavulanic acid; AMK, amikacin; AMX, amoxicillin; ATB, antibiogram; CAZ, ceftazidime; CDA, clindamycin; CHL, chloramphenicol; CIP, ciprofloxacin; Cot, cotrimoxazole; C. spp, *citrobacter* spp; DOX, doxycycline; ERY, erythromycin; I, intermediate; LEV, levofloxacin; M + Ent, malaria positive with enteric infection; M − Ent, malaria negative with enteric infection; NT, not tested; PEN-G, penicillin G; R, resistance; S, sensitive; SXT, trimethoprim/sulphamethoxazole.

**Table 4:** Distribution of coinfected and monoinfected patients with respect to the values of MIC of tested antimalarials.

MIC (μg/mL)	Antimalarial drugs													
	Quinine		Artemether		G-Cospe		Artesunate/amodiaquine		Maalox		Nemether		Surquina 250	
	M + Ent	M − Ent	M + Ent	M − Ent	M + Ent	M − Ent	M + Ent	M − Ent	M + Ent	M − Ent	M + Ent	M − Ent	M + Ent	M − Ent
256	6 (5.66%)	0 (0.00%)	8 (7.55%)	1 (7.69%)	10 (7.87%)	2 (1.15%)	21 (16.54%)	153 (87.93%)	22 (17.32%)	128 (73.56%)	0 (0.00%)	1 (0.57%)	45 (35.43%)	164 (94.25%)
128	14 (13.21%)	2 (14.29%)	8 (7.55)	2 (15.38%)	116 (91.34)	171 (98.28%)	105 (82.68%)	21 (12.05%)	100 (78.74%)	46 (26.44%)	125 (98.4%)	173 (99.43%)	31 (24.41%)	3 (1.72%)
64	33 (31.13%)	6 (42.86%)	89 (83.96%)	10 (76.92%)	0 (0.00%)	1 (0.57%)	0 (0.00%)	0 (0.00%)	5 (3.94%)	0 (0.00%)	1 (0.70%)	0 (0.00%)	50 (39.37%)	7 (4.02%)
32	53 (50.00%)	6 (42.86%)	1 (0.98%)	0 (0.00%)	1 (0.79%)	0 (0.00%)	0 (0.00%)	0 (0.00%)	0 (0.00%)	0 (0.00%)	1 (0.70%)	0 (0.00%)	1 (0.79%)	0 (0.00%)

Abbreviations: M + Ent, malaria positive with enteric infections; M − Ent, malaria negative with enteric infection; MIC, minimum inhibitory concentration.

**Table 5:** Influence of malaria treatment on bacterial resistance.

ATB	Profile	*E. coli*			*E. cloacae*			*K. oxytoca*			*K. pneumoniae*			*Proteus mirabilis*			*Salmonella* spp		
		M + T	M − T	Ent	M + T	M − T	Ent	M + T	M − T	Ent	M + T	M − T	Ent	M + T	M − T	Ent	M + T	M − T	Ent
AKI	R	29 (45.93)	19 (29.68)	14 (21.87)	6 (46.15)	4 (30.76)	3 (23.07)	4 (44.44)	3 (33.33)	2 (22.22)	9 (50.00)	5 (27.77)	4 (22.22)	14 (58.33)	9 (37.50)	8 (33.33)	11 (84.61)	6 (46.15)	5 (38.46)
	S	15 (23.43)	21 (32.81)	26 (40.62)	4 (30.76)	7 (53.84)	6 (46.15)	3 (33.33)	6 (66.66)	7 (77.77)	4 (22.22)	13 (72.22)	11 (61.11)	8 (33.33)	6 (25.00)	7 (29.16)	1 (7.69)	5 (38.46)	4 (30.76)
	I	20 (31.25)	24 (62.50)	14 (21.87)	3 (23.07)	2 (15.38)	4 (30.76)	2 (22.22)	0 (0.00)	0 (0.00)	5 (27.77)	0 (0.00)	3 (16.66)	2 (8.33)	9 (37.50)	9 (37.50)	1 (7.69)	2 (15.38)	4 (30.76)

AMX	R	28 (43.75)	22 (34.37)	16 (57.99)	7 (53.84)	4 (30.76)	4 (30.76)	7 (77.77)	5 (55.55)	3 (33.33)	6 (33.33)	6 (33.33)	5 (27.77)	9 (37.50)	10 (41.66)	6 (25.00)	6 (46.15)	7 (53.84)	6 (46.15)
	S	24 (37.50)	23 (35.93)	26 (40.62)	5 (38.46)	8 (61.53)	5 (38.46)	1 (11.11)	0 (0.00)	4 (44.44)	8 (44.44)	7 (38.88)	4 (22.22)	7 (29.16)	8 (33.33)	10 (41.66)	5 (38.46)	4 (30.76)	3 (23.07)
	I	17 (26.56)	19 (29.68)	22 (34.37)	1 (7.69)	1 (7.69)	4 (30.76)	1 (11.11)	4 (44,44)	2 (22.22)	5 (27.77)	5 (27.77)	4 (22.22)	8 (33.33)	6 (25.00)	8 (33.33)	2 (15.38)	2 (15.38)	4 (30.76)

CIP	R	27 (42.18)	17 (26.56)	9 (14.06)	7 (53.84)	6 (46.15)	7 (53.84)	5 (55.55)	2 (22.22)	0 (0.00)	9 (50.00)	4 (22.22)	6 (33.33)	8 (33.33)	7 (29.16)	8 (33.33)	5 (38.46)	4 (30.76)	4 (30.76)
	S	24 (37.50)	31 (48.43)	26 (40.62)	4 (30.76)	3 (23.07)	3 (23.07)	3 (33.33)	5 (55.55)	6 (66.66)	2 (11.11)	5 (27.77)	8 (44.44)	6 (25.00)	9 (37.50)	9 (37.50)	8 (61.53)	5 (38.46)	7 (53.84)
	I	13 (20.31)	16 (25.00)	29 (45.31)	2 (15.38)	4 (30.76)	3 (23.07)	1 (11.11)	2 (22.22)	3 (33.33)	7 (38.88)	9 (50.00)	4 (22.22)	10 (41.66)	8 (33.33)	7 (29.16)	0 (0.00)	4 (30.76)	2 (15.38)

CHL	R	32 (50.00)	19 (29.68)	21 (32.81)	4 (30.76)	5 (38.46)	6 (46.15)	6 (66.66)	6 (66.66)	5 (55.55)	7 (38.88)	7 (38.88)	5 (27.77)	15 (62.50)	6 (25.00)	6 (25.00)	6 (46.15)	5 (38.46)	3 (23.07)
	S	15 (23.43)	31 (48.43)	18 (28.12)	6 (46.15)	5 (38.46)	2 (15.38)	2 (22.22)	3 (33.33)	2 (22.22)	5 (27.77)	0 (0.00)	4 (22.22)	8 (33.33)	12 (50.00)	7 (29.16)	4 (30.76)	6 (46.15)	0 (0.00)
	I	17 (26.56)	14 (21.87)	25 (39.06)	3 (23.07)	3 (23.07)	5 (38.46)	1 (11.11)	0 (0.00)	2 (22.22)	6 (33.33)	11 (61.11)	9 (50.00)	2 (8.33)	6 (25.00)	11 (45.83)	3 (23.07)	2 (15.38)	10 (76.92)

ERY	R	38 (59.37)	21 (32.81)	17 (26.56)	3 (23.07)	1 (7.69)	1 (7.69)	5 (55.55)	5 (55.55)	2 (22.22)	8 (44.44)	5 (27.77)	9 (50.00)	9 (37.50)	9 (37.50)	7 (29.16)	9 (69.23)	7 (53.84)	8 (61.53)
	S	16 (25.00)	25 (39.06)	19 (29.68)	5 (38.46)	3 (23.07)	6 (46.15)	4 (44.44)	1 (11.11)	4 (44.44)	7 (38.88)	9 (50.00)	6 (33.33)	12 (50.00)	10 (41.66)	8 (33.33)	4 (30.76)	6 (46.15)	4 (30.76)
	I	10 (15.62)	18 (28.12)	28 (43.75)	5 (38.46)	9 (69.23)	6 (46.15)	0 (0.00)	2 (22.22)	3 (33.33)	3 (16.66)	4 (22.22)	3 (16.66)	3 (12.50)	5 (20.83)	9 (37.50)	0 (0.00)	0 (0.00)	1 (7.69)

COT	R	16 (25.00)	6 (9.37)	8 (12.50)	6 (46.15)	3 (23.07)	2 (15.38)	6 (66.66)	8 (88.88)	6 (66.66)	6 (33.33)	5 (27.77)	4 (22.22)	9 (37.50)	8 (33.33)	6 (25.00)	5 (38.46)	4 (30.76)	5 (38.46)
	S	11 (17.18)	29 (45.31)	18 (28.12)	4 (30.76)	2 (15.38)	8 (61.53)	2 (22.22)	1 (11.11)	2 (22.22)	7 (38.88)	4 (22.22)	5 (27.77)	12 (50.00)	9 (37.50)	10 (41.66)	3 (23.07)	0 (0.00)	5 (38.46)
	I	37 (57.81)	29 (45.31)	38 (59.37)	3 (23.07)	8 (61.53)	3 (23.07)	1 (11.11)	0 (0.00)	1 (11.11)	5 (27.77)	9 (50.00)	9 (50.00)	3 (12.50)	7 (29.16)	8 (33.33)	5 (38.46)	9 (69.23)	3 (23.07)

CAZ	R	15 (23.43)	12 (18.75)	19 (29.68)	5 (38.46)	2 (15.38)	3 (23.07)	4 (44.44)	4 (44.44)	3 (33.33)	10 (55.55)	5 (27.77)	2 (11.11)	8 (33.33)	7 (29.16)	8 (33.33)	10 (76.92)	6 (46.15)	7 (53.84)
	S	22 (34.37)	19 (29.68)	24 (37.50)	4 (30.76)	6 (46.15)	4 (30.76)	2 (22.22)	0 (0.00)	5 (55.55)	5 (27.77)	9 (50.00)	8 (44.44)	11 (45.83)	7 (29.16)	7 (29.16)	1 (7.69)	5 (38.46)	3 (23.07)
	I	24 (37.50)	33 (51.56)	4 (6.25)	4 (30.76)	5 (38.46)	8 (61.53)	3 (33.33)	5 (55.55)	1 (11.11)	3 (16.66)	4 (22.22)	8 (44.44)	5 (20.83)	10 (41.66)	9 (37.50)	2 (15.38)	2 (15.38)	3 (23.07)

LEV	R	13 (20.31)	9 (14.06)	7 (10.93)	3 (23.07)	2 (15.38)	1 (7.69)	3 (33.33)	2 (22.22)	0 (0.00)	9 (50.00)	5 (27.77)	5 (27.77)	6 (25.00)	7 (29.16)	6 (25.00)	7 (53.84)	5 (38.46)	3 (23.07)
	S	16 (25.00)	19 (29.68)	4 (6.25)	9 (69.23)	7 (53.84)	5 (38.46)	2 (22.22)	4 (44.44)	5 (55.55)	9 (50.00)	8 (44.44)	7 (38.88)	10 (41.66)	11 (45.83)	9 (37.50)	4 (39.76)	6 (46.15)	10 (76.92)
	I	35 (54.68)	36 (56.25)	1 (1.56)	1 (7.69)	4 (30.76)	7 (53.84)	4 (44.44)	3 (33.33)	4 (44.44)	0 (0.00)	4 (22.22)	6 (33.33)	8 (33.33)	8 (33.33)	9 (37.50)	2 (15.38)	2 (15.38)	0 (0.00)

CDA	R	13 (20.31)	17 (26.56)	6 (9.37)	3 (23.07)	2 (15.38)	2 (15.38)	4 (44.44)	3 (33.33)	2 (22.22)	5 (27.77)	6 (33.33)	5 (27.77)	15 (62.50)	10 (41.66)	9 (37.50)	11 (84.61)	7 (53.84)	0 (0.00)
	S	19 (29.68)	21 (32.81)	19 (29.68)	5 (38.46)	8 (61.53)	7 (53.84)	2 (22.22)	0 (0.00)	6 (66.66)	7 (38.88)	5 (27.77)	11 (61.11)	5 (20.83)	10 (41.66)	11 (45.83)	2 (15.38)	5 (38.46)	8 (61.53)
	I	32 (50.00)	26 (40.62)	39 (60.93)	7 (53.84)	3 (23.07)	4 (30.76)	3 (33.33)	6 (66.66)	1 (11.11)	6 (33.33)	7 (38.77)	2 (11.11)	4 (16.66)	4 (16.66)	4 (16.66)	0 (0.00)	1 (7.69)	5 (38.46)

Abbreviations: AKI, amikacin; AMX, amoxicillin; ATB, antibiogram; CAZ, ceftazidime; CDA, clindamycin; CHL, chloramphenicol; CIP, ciprofloxacin; COT, cotrimoxazole; ERY, erythromycin; Ent, enteric infections; LEV, levofloxacin; M + T, malaria positive and under treatment; M − T, malaria positive not under medications; R, resistance.

## Data Availability

The data used to support the findings of this study are included within the supplementary information file.

## Nomenclature

AMC: Amoxicillin/clavulanic acid
AMK: Amikacin
AMX: Amoxicillin
ATB: Antibiogram
CAZ: Ceftazidime
CDA: Clindamycin
CHL: Chloramphenicol
CIP: Ciprofloxacin
CLSI: Clinical and Laboratory Standards Institute
Cot: Cotrimoxazole
CRP: C-reactive protein
DOX: Doxycycline
EDTA: Ethylenediaminetetraacetic acid
EMB: Eosin methylene blue
ERY: Erythromycin
I: Intermediate
LEV: Levofloxacin
MDR: Multidrug resistance
MHA: Mueller–Hinton agar
MIC: Minimum inhibitory concentration
PEN-G: Penicillin
R: Resistance
RBC: Red blood cells
RDT: Rapid diagnostic test
RND: Resistance nodulation cell division
S: Sensitive
SS: Salmonella-Shigella agar
SXT: Trimethoprim/sulphamethoxazole
WBC: White blood cells

## Supporting Information

Supporting information supporting file (DOCs): S1. Antibiotics used and susceptibility ranking; S2. cutoff presented by different bacteria species; S3. culture media and antimicrobials Supporting information supporting file (DOCs): S4. All raw data were generated to support the findings of this study.
