# Supplementary material for: Haematological Profile and Antibiotic Resistance of Bacteria Responsible for Enteric Infections Isolated From Patients Suffering From Malaria and Enteric Infections on Consultation at the Dschang Regional Hospital
Source: Can J Infect Dis Med Microbiol. 2024 Oct 25;2024:3383995. doi: 10.1155/2024/3383995 (PMC11530289; doi:10.1155/2024/3383995)
Supplement: Supporting Information — Additional supporting information can be found online in the Supporting Information section. [file 3383995.f1.zip › Supplementary_file_S2_Raw data_010623.pdf]

Patient	Sex (SS)	Age(Years)	RDT	GE (Np/mm3)	CRP (mg/dl)	*WBCs*	RBCs
**1**	0	22	0	0	0	*5.31*	5.5
**2**	1	19	0	0	0	*5.6*	5.38
**3**	0	40	0	1	48	*5.6*	4.50
**4**	1	51	1	1	48	*2.8*	2.51
**5**	1	24	0	0	0	*4.6*	6.00
**6**	0	22	0	1	0	*5.7*	5.66
**7**	0	20	0	0	0	*5.0*	4.41
**8**	0	23	0	0	0	*5.2*	3.65
**9**	0	39	0	0	0	*7.5*	4.91
**10**	0	21	0	0	48	*5.0*	4.54
**11**	1	53	0	0	0	*11.3*	1.45
**12**	1	33	0	1	0	*4.8*	5.77
**13**	1	50	0	1	96	*5.0*	4.76
**14**	0	28	0	0	0	*6.4*	4.35
**15**	0	42	0	1	48	*6.1*	2.4
**16**	0	53	0	0	0	*5.1*	4.90
**17**	1	23	0	0	0	*5.5*	5.37
**18**	1	40	0	1	24	*9.9*	5.99
**19**	0	50	0	0	0	*4.7*	3.98
**20**	0	50	0	0	0	*4.0*	4.3
**21**	0	26	1	1	24	*6.7*	4.84
**22**	1	53	1	0	0	*2.8*	1.60
**23**	0	50	0	1	48	*3.0*	2.04
**24**	0	22	1	1	0	*5.9*	4.73
**25**	1	7	0	0	0	*5.4*	4.10
**26**	0	23	0	0	0	*7.2*	4.63
**27**	1	19	0	1	0	*24.4*	4.01
**28**	1	32	0	0	24	*9.6*	4.88
**29**	1	50	0	0	48	*3.6*	5.10
**30**	1	24	0	0	24	*4.2*	4.84
**31**	0	32	0	0	0	*3.7*	4.68
**32**	1	19	0	0	24	*4.6*	4.75
**33**	0	38	0	0	24	*4.3*	4.84
**34**	1	65	1	1	0	*4.7*	2.64
**35**	0	17	0	1	0	*6.1*	4.98
**36**	0	24	0	0	12	*5.8*	4.61
**37**	0	27	0	0	0	*7.5*	4.34
**38**	0	30	0	0	0	*4.1*	5.04
**39**	1	51	1	0	0	*2.8*	2.46
**40**	0	60	0	0	0	*4.0*	4.75
**41**	0	20	0	0	0	*6.6*	4.09
**42**	0	19	0	0	0	*3.0*	4.20
**43**	1	36	0	1	12	*8.2*	5.23
**44**	0	23	0	1	0	*20.4*	4.12
**45**	1	55	1	1	48	*7.3*	4.09
**46**	0	35	0	0	0	*4.0*	3.93

**47**	1	65	0	1	0	*7.2*	3.68
**48**	0	28	0	1	12	*7.0*	3.99
**49**	1	31	0	0	0	*11.4*	6.66
**50**	1	28	0	0	0	*7.0*	5.21
**51**	1	41	0	1	48	*4.4*	6.08
**52**	1	36	0	0	0	*6.6*	4.51
**53**	1	28	0	0	0	*8.2*	5.24
**54**	1	10	1	1	48	*7.3*	2.57
**55**	0	18	0	1	0	*6.2*	5.46
**56**	0	39	0	0	0	*5.3*	5.88
**57**	0	19	0	1	48	*5.9*	4.40
**58**	1	15	1	1	48	*6.3*	3.65
**59**	0	23	0	1	6	*4.5*	3.75
**60**	0	26	0	0	0	*5.2*	4.94
**61**	0	32	1	1	24	*2.6*	2.28
**62**	1	35	0	1	48	*6.8*	5.02
**63**	0	26	0	1	24	*5.5*	4.62
**64**	0	21	0	0	0	*2.8*	3.91
**65**	1	10	0	1	48	*5.4*	3.78
**66**	1	12	0	1	24	*11.5*	4.36
**67**	0	21	0	0	0	*3.7*	4.56
**68**	0	36	1	1	48	*4.8*	4.37
**69**	1	13	0	0	0	*5.6*	5.20
**70**	0	23	0	0	12	*3.4*	4.34
**71**	0	54	1	1	48	*6.6*	3.32
**72**	0	58	0	0	0	*6.1*	4.86
**73**	0	27	0	0	0	*4.3*	5.10
**74**	0	45	0	1	0	*6.6*	5.18
**75**	0	20	0	0	0	*4.9*	4.59
**76**	0	27	0	0	0	*7.0*	4.45
**77**	0	20	0	0	0	*4.3*	3.90
**78**	0	19	0	0	0	*11.1*	2.45
**79**	1	39	0	1	0	*3.0*	5.03
**80**	1	51	1	1	24	*2.0*	2.76
**81**	0	60	0	0	0	*5.0*	4.26
**82**	0	40	0	0	0	*7.3*	4.25
**83**	0	31	0	0	0	*5.3*	5.03
**84**	1	22	0	0	0	*6.0*	5.66
**85**	0	21	0	1	6	*11.5*	4.55
**86**	0	58	0	0	0	*3.1*	4.35
**87**	0	19	0	0	0	*5.2*	4.45
**88**	1	16	1	1	24	*2.5*	3.54
**89**	0	57	0	0	0	*3.7*	4.50
**90**	0	32	0	0	0	*4.3*	4.96
**91**	0	51	0	0	0	*4.4*	5.43
**92**	0	22	0	0	0	*8.6*	4.45
**93**	1	49	1	1	48	*2.6*	4.77
**94**	1	56	0	0	0	*3.8*	4.92
**95**	1	6	0	0	0	*6.9*	3.05
**96**	0	22	1	1	48	*7.1*	2.4

**97**	0	21	0	0	0	*6.4*	4.71
**98**	1	15	0	0	0	*4.6*	5.88
**99**	0	19	0	0	0	*4.3*	6.03
**100**	0	22	0	0	0	*5.3*	5.51
**101**	1	5	0	0	12	*3.9*	4.41
102	0	53	0	1	0	*6.9*	3.93
103	0	42	1	1	0	*4.4*	4.66
104	0	22	0	1	0	*5.2*	3.03
105	1	8	0	1	0	*6.6*	5.66
106	1	55	1	1	0	*3.5*	2.54
107	0	25	0	0	0	*4.9*	5.43
108	0	11	0	0	0	*4.5*	4.92
109	1	19	0	1	48	*6.6*	3.5
110	0	32	0	1	48	*2.6*	4.4
**111**	1	21	0	0	0	*4.9*	1.45
**112**	1	1	0	1	0	*4.5*	4.88
**113**	0	28	0	1	192	*6.9*	3.93
**114**	0	34	0	1	192	*4.4*	6.66
**115**	0	22	0	0	0	*4.2*	5.03
**116**	0	1	0	0	12	*6.6*	5.66
**117**	1	1	0	1	48	*4.5*	4.54
**118**	1	6	0	1	12	*3.9*	5.43
**119**	1	23	0	0	48	*5.5*	4.92
**120**	0	50	0	0	48	*5.4*	6.5
**121**	0	19	0	0	0	*6.6*	4.4
**122**	1	21	1	1	0	*7.4*	4.0
**123**	1	49	1	1	6	*6.9*	3.05
**124**	1	23	0	1	0	*7.1*	4.4
**125**	0	11	0	0	0	*6.4*	4.71
**126**	0	22	1	1	0	*4.6*	3.88
**127**	0	59	0	0	0	*4.3*	6.03
**128**	1	20	0	0	0	*5.3*	5.51
**129**	0	57	1	1	12	*3.9*	4.41
**130**	0	57	1	1	48	*4.9*	1.45
**131**	0	22	0	0	24	*2.5*	4.88
**132**	0	63	0	1	0	*6.9*	3.93
**133**	0	22	0	1	0	*4.4*	4.66
**134**	0	30	0	1	0	*4.2*	2.03
**135**	1	6	0	1	0	*6.6*	5.66
**136**	0	65	0	1	0	*4.5*	2.54
**137**	1	25	0	0	0	*3.9*	5.43
**138**	1	7	0	0	0	*5.5*	4.92
**139**	0	17	0	1	48	*5.4*	3.5
**140**	0	22	0	1	48	*2.6*	4.4
**141**	0	75	0	0	0	*6.3*	4.0
**142**	1	49	0	1	0	*5.5*	4.1
**143**	1	18	0	0	0	*7.6*	4.2
**144**	0	24	0	0	0	*9.4*	4.3
**145**	1	58	0	0	0	*7.8*	4.4
**146**	1	16	0	0	48	*4.9*	4.5

**147**	0	24	0	0	0	*5.10*	4.6
**148**	0	9	0	1	0	*7.11*	4.7
**149**	0	25	0	1	48	*3.18*	1.8
**150**	0	20	0	0	0	*7.13*	4.9
**151**	1	22	0	0	0	*9.12*	4.10
**152**	0	15	0	0	0	*7.15*	4.11
**153**	0	27	0	1	0	*6.16*	4.12
**154**	0	25	0	0	24	*5.16*	3.13
**155**	0	23	0	0	48	*9.18*	4.14
**156**	0	7	0	1	0	*2.10*	4.15
**157**	0	20	0	0	0	*6.20*	4.16
**158**	1	51	0	1	12	*2.21*	317
**159**	1	26	1	1	0	*2.22*	4.18
**160**	0	19	0	0	48	*7.23*	4.19
**161**	0	21	0	0	0	*7.24*	4.20
**162**	1	1	0	1	0	*2.25*	3.21
**163**	1	28	0	0	12	*5.26*	4.22
**164**	1	34	0	0	0	*7.27*	6.23
**165**	1	22	0	0	0	*7.28*	2.24
**166**	1	1	0	0	0	*8.29*	8.25
**167**	0	1	0	0	0	*7.30*	4.26
**168**	0	6	0	0	0	*7.31*	4.27
**169**	0	23	0	1	0	*6.32*	5.28
**170**	1	50	0	1	0	*2.33*	3.29
**171**	0	19	0	0	24	*6.34*	4.30
**172**	0	21	0	0	0	*7.35*	4.31
**173**	0	49	0	0	0	*5.36*	6.72
**174**	0	23	0	0	0	*7.37*	4.32
**175**	0	11	0	0	48	*4.32*	5.34
**176**	0	22	0	0	0	*7.39*	4.35
**177**	1	59	0	0	0	*6.40*	7.36
**178**	0	20	0	1	0	*5.41*	4.37
**179**	1	57	0	1	0	*2.40*	2.38
**180**	1	57	0	1	12	*3.43*	4.39
**181**	0	22	1	0	0	*8.44*	2.40
**182**	0	63	0	1	0	*7.45*	4.41
**183**	0	22	1	1	192	*2.42*	3.46
**184**	1	30	0	0	0	*4.47*	4.43
**185**	1	6	0	0	0	*7.48*	3.04
**186**	0	65	1	1	48	*2.49*	3.49
**187**	1	25	0	0	0	*7.50*	4.46
**188**	0	35	0	0	0	*5.21*	2.33
**189**	1	7	0	0	0	*7.51*	5.44
**190**	0	22	0	1	0	*2.56*	2.48
**191**	0	19	0	0	0	*3.53*	6.49
**192**	0	40	0	0	48	*6.50*	4.60
**193**	0	28	1	1	48	*7.55*	4.51
**194**	1	60	1	1	48	*2.51*	3.57
**195**	0	22	0	1	48	*7.57*	3.53
**196**	0	20	0	0	0	*4.52*	4.52

**197**	0	23	0	1	0	*7.59*	3.55
**198**	0	39	0	1	0	*2.60*	5.56
**199**	0	21	1	1	0	*2.61*	2.57
**200**	0	53	0	0	0	*6.69*	4.50
**201**	1	33	0	0	48	*7.63*	3.59
**202**	1	50	0	0	48	*4.64*	7.60
**203**	0	28	1	1	12	*8.64*	4.61
**204**	0	53	1	1	12	*2.66*	2.66
**205**	1	23	0	0	0	*7.67*	4.63
**206**	1	40	0	0	0	*5.68*	7.64
**207**	1	1	0	1	0	*7.25*	5.21
**208**	1	28	0	0	12	*5.26*	4.22
**209**	1	34	0	0	0	*7.27*	6.23
**210**	1	22	0	0	0	*7.28*	4.24
**211**	1	1	0	0	0	*8.29*	8.25
**212**	0	1	0	0	0	*7.30*	4.26
**213**	0	6	0	0	0	*7.31*	4.27
**214**	0	23	1	1	0	*2.32*	2.28
**215**	1	50	0	1	0	*7.33*	4.29
**216**	0	19	0	0	24	*6.34*	4.30
**217**	0	21	0	0	0	*7.35*	4.31
**218**	0	49	0	0	0	*5.36*	6.72
**219**	1	59	0	0	0	*6.40*	5.36
**220**	0	20	1	1	0	*2.41*	2.37
**221**	1	57	0	1	0	*6.42*	4.38
**222**	1	57	1	1	12	*7.43*	339
**223**	0	22	0	0	0	*8.44*	6.40
**224**	0	63	0	1	0	*7.45*	4.41
**225**	0	22	1	1	192	*2.46*	6.46
**226**	1	30	0	0	0	*4.47*	4.43
**227**	1	6	0	0	0	*7.48*	3.04
**228**	0	65	1	1	48	*5.49*	2.49
**229**	1	25	0	0	0	*7.50*	4.46
**230**	1	7	0	0	0	*7.51*	5.44
**231**	0	22	1	1	0	*3.52*	7.48
**232**	0	19	0	0	0	*3.53*	6.49
**233**	0	40	0	0	48	*6.50*	4.60
**234**	0	28	0	1	48	*2.55*	2.51
**235**	0	22	0	0	0	*8.6*	4.45
**236**	1	49	1	1	48	*2.6*	4.77
**237**	1	56	0	0	0	*3.8*	4.92
**238**	1	6	0	0	0	*6.9*	4.05
**239**	0	22	0	1	48	*3.1*	3.4
**240**	0	21	0	0	0	*6.4*	4.71
**241**	1	15	0	0	0	*4.6*	5.88
**242**	0	19	0	0	0	*4.3*	6.03
**243**	0	22	0	0	0	*5.3*	5.51
**244**	1	5	0	0	12	*3.9*	4.41
**245**	1	21	0	0	0	*4.9*	1.45
**246**	1	1	1	1	0	*2.5*	2.88

**247**	0	58	1	1	192	*2.9*	2.93
**248**	0	54	1	1	192	*2.4*	2.66
**249**	0	22	0	0	0	*4.2*	5.03
**250**	0	1	0	0	12	*6.6*	5.66
**251**	1	1	0	1	48	*4.5*	4.54
**252**	1	6	1	1	12	*3.9*	5.43
**253**	1	23	0	0	48	*5.5*	4.92
**254**	0	50	0	1	48	*5.4*	6.5
**255**	0	19	0	0	48	*7.23*	4.19
**256**	0	21	0	0	0	*4.24*	4.20
**257**	1	1	0	1	0	*7.25*	5.21
**258**	1	28	0	0	12	*5.26*	4.22
**259**	1	34	0	0	0	*7.27*	6.23
**260**	1	22	0	0	0	*7.28*	4.24
**261**	1	1	0	0	0	*8.29*	8.25
**262**	0	1	0	0	0	*7.30*	6.26
**263**	1	56	0	0	0	*3.8*	4.92
**264**	1	6	0	0	0	*6.9*	4.05
**265**	0	22	1	1	48	*3.1*	2.40
**266**	0	21	0	0	0	*6.4*	4.71
**267**	1	15	0	0	0	*4.6*	5.88
**268**	0	19	0	0	0	*4.3*	6.03
**269**	0	22	0	1	0	*5.3*	5.51
**270**	1	5	0	0	12	*3.9*	4.41
**271**	1	21	0	0	0	*4.9*	1.45
**272**	1	5	1	1	0	*4.5*	4.88
**273**	0	28	1	1	192	*6.9*	3.93
**274**	0	74	1	1	192	*8.4*	6.66
**275**	0	22	0	0	0	*4.2*	5.03
**276**	1	24	0	0	0	*4.6*	6.00
**277**	0	52	0	1	0	*2.7*	5.66
**278**	0	20	0	0	0	*5.0*	4.41
**279**	0	23	0	0	0	*5.2*	3.65
**280**	0	39	0	0	0	*7.5*	4.91
**281**	0	21	0	1	48	*5.0*	4.54
**282**	1	53	0	0	0	*11.3*	1.45
**283**	1	33	0	1	0	*2.8*	5.77
**284**	1	50	0	1	96	*2.0*	4.76
**285**	0	9	0	1	0	*7.11*	4.7
**286**	1	65	0	1	48	*1.18*	2.8
**287**	0	20	0	0	0	*7.13*	4.9
**288**	0	55	0	1	48	*2.&(*	3.8
**289**	0	22	0	0	0	*9.12*	4.10
**290**	0	15	0	0	0	*7.15*	4.11
**291**	1	27	0	1	0	*6.16*	4.12
**292**	0	25	0	0	24	*5.16*	3.13
**293**	0	23	0	0	48	*7.17*	4.14
**294**	1	7	0	1	0	*2.10*	6.15
**295**	0	60	0	0	0	*2.20*	4.16
**296**	1	51	0	1	12	*2.21*	6.17

**297**	1	26	1	1	0	*4.22*	2.18
**298**	1	6	0	1	0	*2.6*	1.66
**299**	0	65	0	1	0	*4.5*	2.54
**300**	1	25	0	0	0	*3.9*	5.43
**301**	0	61	1	1	0	*2.61*	2.57

HGB	*HCT*	MCV	MCH	MCHC	PLT	LY	MO
11.3	*35.0*	65.9	21.3	31.3	364	37.2	3.5
12.8	*39.1*	72.7	23.8	32.7	272	23.6	3
12.5	*37.3*	82.9	27.8	33.5	235	22.2	2.5
10.4	*32.6*	80.9	27.9	34.5	114	18.9	1.9
16.0	*47.5*	79.2	26.7	33.7	241	45.9	5.3
13.3	*43.2*	76.3	23.5	30.8	380	15.4	3.7
12.5	*38.6*	87.5	28.3	34.4	253	39.1	2.4
9.9	*30.2*	82.7	27.1	32.8	325	38.4	2.4
13.6	*43.7*	89.0	27.7	31.1	391	24.1	2.5
12.2	*38.6*	85.0	26.9	31.6	202	28.2	4.5
5.0	*14.3*	98.6	34.5	35.0	145	6.7	2.2
14.4	*44.7*	77.5	25.0	32.2	249	38.6	2.4
13.3	*40.9*	85.9	27.1	32.5	119	27.1	6.4
13.1	*40.1*	92.2	30.1	32.7	185	25.5	5.9
11.8	*31.9*	71.3	33.5	33.9	394	17.6	2.5
12.0	*38.0*	77.6	24.5	31.6	227	31.6	3.1
14.1	*42.8*	79.7	26.3	32.9	218	35.0	2.6
15.7	*47.6*	79.5	26.2	33.0	191	9.8	2.2
10.4	*31.6*	79.4	26.1	32.9	293	37.5	4.4
11.6	*36.1*	87.4	28.1	32.1	269	36.2	4.4
11.8	*37.1*	77.1	24.4	31.6	440	40.4	3.8
10.5	*35.9*	80.2	25.9	32.3	109	37.7	7.3
10.3	*34.5*	85.4	28.0	32.8	119	14.8	3.1
13.2	*40.8*	86.3	27.9	32.4	196	25.8	5.1
11.4	*33.3*	81.2	27.8	34.2	283	35.7	3.3
13.3	*39.9*	86.2	28.7	24.3	172	21.9	3.0
9.5	*28.1*	70.1	23.7	23.8	259	7.2	2.8
13.8	*40.7*	83.4	28.3	33.9	255	7.4	1.6
13.4	*40.7*	79.8	26.3	32.9	219	32.9	4.5
12.8	*37.9*	78.3	26.4	33.8	126	22.4	5.8
13.5	*40.0*	85.5	28.8	33.8	203	45.4	4.1
13.1	*39.4*	82.9	27.6	33.2	136	15.1	2.1
13.2	*39.0*	80.6	27.3	33.8	222	45.2	3.5
11.1	*36.9*	83.2	28.5	34.3	111	32.6	3.9
14.1	*40.9*	82.1	28.3	34.5	281	28.3	2.7
13.2	*39.7*	86.1	28.6	33.2	231	16.6	3.7
11.7	*35.4*	81.6	27.0	33.1	348	27.2	3.6
13.7	*42.3*	83.9	27.2	32.4	248	39.0	3.4
13.3	*34.3*	81.1	24.4	30.0	60	22.4	10.0
11.2	*38.3*	80.6	23.6	29.2	213	34.3	3.9
8.9	*31.1*	76.0	21.8	28.6	179	35.0	3.0
11.1	*34.4*	81.9	26.4	32.2	202	34.0	4.1
14.1	*41.7*	79.7	27.0	33.8	270	10.7	3.3
12.6	*36.1*	87.6	30.6	34.9	191	13.2	1.9
12.1	*34.9*	85.3	29.6	34.7	110	15.8	2.5
11.9	*35.9*	91.3	30.3	33.1	100	49.0	4.0

10.0	*29.6*	80.4	27.2	33.8	220	16.6	4.4
10.5	*31.7*	79.4	26.3	33.1	108	12.1	3.5
14.5	*45.0*	67.6	21.8	32.2	182	5.6	1.8
14.6	*42.9*	82.3	28.0	34.0	115	26.0	2.9
15.3	*47.2*	77.6	25.2	32.4	166	20.4	2.3
13.2	*39.4*	87.4	29.3	33.5	181	24.2	3.3
14.8	*43.3*	82.6	28.2	34.2	228	11.4	2.7
13.4	*19.0*	73.9	24.9	26.7	100	35.3	5.5
13.2	*39.4*	72.2	24.2	33.5	268	42.2	3.3
17.4	*52.4*	89.4	29.7	23.2	67	20.4	2.9
11.7	*35.2*	80.0	26.6	33.2	285	35.1	5.3
10.1	*31.2*	85.5	27.7	32.4	143	50.6	3.9
12.7	*38.9*	81.9	26.7	22.6	173	23.5	4.0
12.4	*38.3*	77.5	25.1	32.4	259	44.8	3.6
11.3	*33.3*	77.8	26.4	33.9	100	17.0	4.6
11.8	*35.1*	69.9	23.5	33.6	224	20.9	2.7
11.5	*34.4*	74.5	24.9	33.4	134	29.5	3.9
10.6	*31.1*	79.5	22.1	34.1	194	31.1	2.8
10.1	*30.0*	79.4	26.7	33.7	74	26.8	4.7
11.4	*34.4*	78.9	26.1	33.1	244	19.2	3.1
12.8	*37.0*	81.1	28.1	34.6	167	34.5	3.7
12.1	*36.7*	84.0	27.7	33.0	65	18.7	2.8
13.1	*39.7*	76.3	25.2	33.0	351	41.2	2.3
12.0	*35.9*	82.7	27.6	33.4	220	16.7	3.0
11.7	*34.1*	78.9	27.1	34.3	115	12.1	1.8
13.0	*38.8*	79.8	26.7	33.5	211	38.2	2.6
12.2	*36.7*	72.0	23.9	33.2	207	32.5	3.2
14.7	*45.8*	88.4	28.4	32.1	235	31.9	4.9
12.8	*38.0*	82.8	27.9	33.7	240	32.1	3.2
12.5	*37.7*	84.7	28.1	33.2	238	40.1	2.7
9.5	*29.2*	74.9	24.4	25.5	304	31.2	4.8
7.5	*21.6*	88.2	30.6	34.7	294	17.5	2.8
13.9	*40.5*	80.5	27.6	34.3	191	34.2	4.7
12.0	*21.0*	76.1	21.4	33.3	120	27.2	3.5
11.6	*34.6*	81.2	27.2	33.5	238	25.5	4.2
11.8	*34.6*	81.4	24.8	34.1	243	31.5	3.9
13.1	*40.0*	79.5	26.0	32.8	202	35.5	3.4
15.2	*46.3*	81.8	26.9	32.8	214	23.4	3.2
12.2	*36.5*	80.2	26.8	33.4	268	24.4	1.4
12.4	*36.7*	84.4	28.5	33.8	227	44.7	3.1
12.8	*37.7*	84.7	28.8	34.0	286	40.6	4.0
12.6	*28.4*	84.6	27.8	32.8	60	25.9	4.3
11.6	*35.2*	78.2	25.8	33.0	247	33.4	3.2
12.7	*37.8*	76.2	25.6	33.6	194	47.4	3.1
14.5	*43.5*	80.1	26.7	33.3	209	28.9	3.5
13.6	*40.7*	91.5	30.6	33.4	272	38.1	3.4
13.4	*40.0*	83.9	28.1	33.5	70	27.4	5.2
12.6	*40.9*	83.1	25.6	30.8	232	44.8	3.4
10.1	*30.1*	74.3	24.9	33.6	336	27.0	2.0
10.8	*31.9*	72.3	24.5	33.9	394	15.6	2.5

13.1	*38.3*	81.3	27.8	34.2	155	17.4	1.5
14.8	*45.0*	76.5	25.2	32.9	302	38.7	4.3
14.0	*43.2*	71.6	23.2	32.4	<u>183</u>	30.4	2.1
12.8	*39.1*	72.7	23.8	32.7	<u>272</u>	23.6	1.9
12.5	*37.3*	82.9	27.8	33.5	235	22.2	2.5
13.3	*39.9*	76.2	28.7	33.3	162	31.9	2.0
9.5	*28.1*	70.1	23.7	33.8	259	7.2	2.8
10.8	*40.7*	83.4	28.3	33.9	155	7.4	1.6
13.4	*40.7*	89.8	26.3	32.9	219	32.9	4.5
12.8	*37.9*	78.3	26.4	33.8	146	22.4	4.8
13.5	*40.0*	85.5	28.8	33.8	203	45.4	4.1
13.1	*39.4*	82.9	27.6	23.2	136	15.1	2.1
13.2	*29.0*	80.6	27.3	33.8	122	45.2	3.5
16.1	*26.9*	83.2	28.5	34.3	<u>211</u>	32.6	3.9
15.4	*44.6*	80.9	27.9	34.5	<u>214</u>	18.9	1.9
16.0	*47.5*	79.2	26.7	33.7	<u>241</u>	45.9	5.3
13.3	*43.2*	76.3	23.5	30.8	380	15.4	3.7
12.5	*38.6*	87.5	28.3	32.4	253	39.1	2.4
9.9	*30.2*	82.7	27.1	32.8	325	38.4	2.4
13.6	*43.7*	89.0	27.7	31.1	391	24.1	2.5
12.2	*38.6*	85.0	26.9	31.6	202	28.2	4.5
5.0	*14.3*	98.6	34.5	35.0	145	6.7	2.2
14.4	*44.7*	77.5	25.0	32.2	249	38.6	2.4
13.3	*40.9*	85.9	27.1	32.5	119	27.1	6.4
13.1	*40.1*	92.2	30.1	32.7	185	25.5	5.9
12.0	*38.0*	77.6	24.5	31.6	127	31.6	3.1
14.1	*42.8*	79.7	26.3	32.9	218	35.0	2.6
11.7	*47.6*	79.5	26.2	33.0	191	9.8	2.2
10.4	*31.6*	79.4	26.1	32.9	293	37.5	4.4
11.6	*36.1*	87.4	28.1	22.1	269	36.2	4.4
9.6	*37.1*	77.1	24.4	31.6	440	40.4	3.8
14.5	*44.9*	80.2	25.9	32.3	299	37.7	7.3
11.3	*34.5*	85.4	28.0	32.8	149	14.8	3.1
13.2	*30.8*	86.3	27.9	32.4	196	25.8	5.1
11.4	*33.3*	81.2	27.8	34.2	283	35.7	3.3
13.3	*39.9*	86.2	28.7	33.3	172	21.9	3.0
9.5	*28.1*	70.1	23.7	33.8	259	7.2	2.8
10.8	*40.7*	83.4	28.3	33.9	255	7.4	1.6
13.4	*40.7*	79.8	26.3	32.9	219	32.9	4.5
12.8	*37.9*	78.3	26.4	33.8	126	22.4	5.8
13.5	*40.0*	85.5	28.8	33.8	203	45.4	4.1
13.1	*39.4*	82.9	27.6	23.2	136	15.1	2.1
13.2	*29.0*	80.6	27.3	33.8	122	45.2	3.5
16.1	*26.9*	83.2	28.5	34.3	211	32.6	3.9
14.1	*40.9*	82.1	28.3	34.5	281	28.3	2.7
13.2	*39.7*	86.1	28.6	33.2	231	16.6	3.7
11.7	*35.4*	81.6	27.0	33.1	348	27.2	3.6
13.7	*42.3*	83.9	27.2	32.4	248	39.0	3.4
13.3	*44.3*	81.1	24.4	30.0	60	22.4	10.0
11.2	*38.3*	80.6	23.6	29.2	213	34.3	3.9

8.9	*31.1*	76.0	21.8	28.6	179	35.0	3.0
11.1	*34.4*	81.9	26.4	32.2	202	34.0	4.1
14.1	*41.7*	79.7	27.0	33.8	270	10.7	3.3
12.6	*36.1*	87.6	30.6	34.9	191	13.2	1.9
12.1	*34.9*	85.3	29.6	34.7	110	15.8	2.5
11.9	*35.9*	91.3	30.3	33.1	100	49.0	4.0
10.0	*29.6*	80.4	27.2	33.8	220	16.6	4.4
10.5	*31.7*	79.4	26.3	33.1	108	12.1	3.5
14.5	*45.0*	67.6	21.8	32.2	182	5.6	1.8
14.6	*32.9*	82.3	28.0	34.0	115	26.0	2.9
15.3	*47.2*	77.6	25.2	32.4	166	20.4	2.3
13.2	*39.4*	87.4	29.3	33.5	181	24.2	3.3
14.8	*33.3*	82.6	28.2	34.2	128	11.4	2.7
6.4	*19.0*	73.9	24.9	33.7	150	35.3	5.5
13.2	*39.4*	72.2	24.2	27.5	268	42.2	3.3
17.4	*52.4*	89.4	29.7	33.2	67	20.4	2.9
11.7	*35.2*	80.0	26.6	33.2	285	35.1	5.3
10.1	*31.2*	85.5	27.7	32.4	143	50.6	3.9
12.7	*38.9*	81.9	26.7	32.6	173	23.5	4.0
12.4	*38.3*	77.5	25.1	32.4	259	44.8	3.6
6.3	*33.3*	77.8	26.4	33.9	100	17.0	4.6
11.8	*35.1*	69.9	23.5	33.6	224	20.9	2.7
11.5	*34.4*	74.5	24.9	33.4	134	29.5	3.9
10.6	*31.1*	79.5	27.1	34.1	194	31.1	2.8
10.1	*30.0*	79.4	26.7	33.7	74	26.8	4.7
11.4	*34.4*	78.9	26.1	33.1	244	19.2	3.1
6.8	*37.0*	81.1	28.1	34.6	167	34.5	3.7
12.1	*36.7*	84.0	27.7	33.0	65	18.7	2.8
13.1	*39.7*	76.3	25.2	33.0	351	41.2	2.3
12.0	*35.9*	82.7	27.6	33.4	220	16.7	3.0
11.7	*34.1*	78.9	27.1	34.3	215	12.1	1.8
13.0	*38.8*	79.8	26.7	33.5	211	38.2	2.6
12.2	*36.7*	72.0	23.9	33.2	207	32.5	3.2
14.7	*45.8*	88.4	28.4	32.1	235	31.9	4.9
12.8	*38.0*	82.8	27.9	33.7	240	32.1	3.2
12.5	*37.7*	84.7	28.1	33.2	238	40.1	2.7
9.5	*29.2*	74.9	24.4	32.5	304	31.2	4.8
7.5	*21.6*	88.2	30.6	34.7	294	17.5	2.8
13.9	*40.5*	80.5	27.6	34.3	191	34.2	4.7
7.0	*21.0*	76.1	25.4	33.3	180	27.2	3.5
10.6	*34.6*	81.2	27.2	33.5	238	25.5	4.2
6.3	*30.4*	69.4	25.8	30	179	29.2	2.1
11.8	*34.6*	81.4	27.8	34.1	243	31.5	3.9
13.1	*40.0*	79.5	26.0	32.8	102	35.5	3.4
15.2	*46.3*	81.8	26.9	32.8	214	23.4	3.2
13.2	*36.5*	80.2	26.8	33.4	268	24.4	1.4
12.4	*36.7*	84.4	28.5	33.8	227	44.7	3.1
12.8	*37.7*	84.7	28.8	34.0	186	40.6	4.0
7.6	*38.4*	84.6	27.8	32.8	60	25.9	4.3
11.6	*35.2*	78.2	25.8	33.0	247	33.4	3.2

12.7	*37.8*	76.2	25.6	33.6	104	47.4	3.1
14.5	*43.5*	80.1	26.7	33.3	209	28.9	3.5
13.6	*40.7*	91.5	30.6	33.4	272	38.1	3.4
13.4	*40.0*	83.9	28.1	33.5	70	27.4	5.2
12.6	*40.9*	83.1	25.6	30.8	232	44.8	3.4
10.1	*30.1*	74.3	24.9	33.6	336	27.0	2.0
10.8	*31.9*	72.3	24.5	33.9	394	15.6	2.5
13.1	*28.3*	81.3	27.8	34.2	155	17.4	1.5
14.8	*45.0*	76.5	25.2	32.9	302	38.7	4.3
7.0	*43.2*	71.6	23.2	32.4	183	30.4	2.1
17.4	*52.4*	89.4	29.7	33.2	67	20.4	2.9
11.7	*35.2*	80.0	26.6	33.2	285	35.1	5.3
10.1	*31.2*	85.5	27.7	32.4	143	50.6	3.9
12.7	*38.9*	81.9	26.7	32.6	173	23.5	4.0
12.4	*38.3*	77.5	25.1	32.4	259	44.8	3.6
6.3	*33.3*	77.8	26.4	33.9	100	17.0	4.6
11.8	*35.1*	69.9	23.5	33.6	224	20.9	2.7
11.5	*34.4*	74.5	24.9	33.4	134	29.5	3.9
10.6	*31.1*	79.5	27.1	34.1	194	31.1	2.8
10.1	*30.0*	79.4	26.7	33.7	74	26.8	4.7
11.4	*34.4*	78.9	26.1	33.1	244	19.2	3.1
6.8	*37.0*	81.1	28.1	34.6	167	34.5	3.7
11.7	*34.1*	78.9	27.1	34.3	215	12.1	1.8
13.0	*34.8*	79.8	26.7	33.5	211	38.2	2.6
12.2	*36.7*	72.0	23.9	33.2	207	32.5	3.2
14.7	*45.8*	88.4	28.4	32.1	235	31.9	4.9
12.8	*38.0*	82.8	27.9	33.7	240	32.1	3.2
12.5	*37.7*	84.7	28.1	33.2	138	40.1	2.7
9.5	*29.2*	74.9	24.4	32.5	104	31.2	4.8
7.5	*21.6*	88.2	30.6	34.7	294	17.5	2.8
13.9	*30.5*	80.5	27.6	34.3	191	34.2	4.7
7.0	*21.0*	76.1	25.4	33.3	180	27.2	3.5
10.6	*34.6*	81.2	27.2	33.5	238	25.5	4.2
11.8	*34.6*	81.4	27.8	34.1	243	31.5	3.9
13.1	*40.0*	79.5	26.0	32.8	202	35.5	3.4
15.2	*46.3*	81.8	26.9	32.8	214	23.4	3.2
13.2	*36.5*	80.2	26.8	33.4	268	24.4	1.4
12.4	*36.7*	84.4	28.5	33.8	227	44.7	3.1
13.6	*40.7*	91.5	30.6	33.4	272	38.1	3.4
13.4	*30.0*	83.9	28.1	33.5	70	27.4	5.2
12.6	*40.9*	83.1	25.6	30.8	232	44.8	3.4
10.1	*30.1*	74.3	24.9	33.6	336	27.0	2.0
10.8	*31.9*	72.3	24.5	33.9	94	15.6	2.5
13.1	*38.3*	81.3	27.8	34.2	155	17.4	1.5
14.8	*45.0*	76.5	25.2	32.9	302	38.7	4.3
14.0	*43.2*	71.6	23.2	32.4	183	30.4	2.1
12.8	*39.1*	72.7	23.8	32.7	272	23.6	3
12.5	*37.3*	82.9	27.8	33.5	235	22.2	2.5
15.4	*44.6*	80.9	27.9	34.5	214	18.9	1.9
11.0	*27.5*	79.2	26.7	33.7	141	45.9	5.3

13.3	*43.2*	76.3	23.5	30.8	380	15.4	3.7
12.5	*28.6*	87.5	28.3	32.4	253	39.1	2.4
9.9	*30.2*	82.7	27.1	32.8	325	38.4	2.4
13.6	*43.7*	89.0	27.7	31.1	391	24.1	2.5
12.2	*38.6*	85.0	26.9	31.6	202	28.2	4.5
5.0	*14.3*	98.6	34.5	35.0	145	6.7	2.2
14.4	*44.7*	77.5	25.0	32.2	249	38.6	2.4
13.3	*40.9*	85.9	27.1	32.5	119	27.1	6.4
6.4	*19.0*	73.9	24.9	33.7	150	35.3	5.5
13.2	*39.4*	72.2	24.2	33.5	268	42.2	3.3
17.4	*52.4*	89.4	29.7	33.2	67	20.4	2.9
11.7	*35.2*	80.0	26.6	33.2	285	35.1	5.3
10.1	*31.2*	85.5	27.7	32.4	143	50.6	3.9
12.7	*38.9*	81.9	26.7	32.6	173	23.5	4.0
12.4	*38.3*	77.5	25.1	32.4	259	44.8	3.6
6.3	*33.3*	77.8	26.4	33.9	100	17.0	4.6
12.6	*40.9*	83.1	25.6	30.8	232	44.8	3.4
10.1	*30.1*	74.3	24.9	33.6	336	27.0	2.0
10.8	*31.9*	72.3	24.5	33.9	94	15.6	2.5
13.1	*38.3*	81.3	27.8	34.2	155	17.4	1.5
14.8	*45.0*	76.5	25.2	32.9	302	38.7	4.3
14.0	*43.2*	71.6	23.2	27.4	183	30.4	2.1
12.8	*39.1*	72.7	23.8	32.7	272	23.6	3
12.5	*37.3*	82.9	27.8	33.5	235	22.2	2.5
15.4	*44.6*	80.9	27.9	34.5	214	18.9	1.9
12.0	*27.5*	79.2	26.7	33.7	241	45.9	5.3
13.3	*43.2*	76.3	23.5	30.8	380	15.4	3.7
12.5	*28.6*	87.5	28.3	32.4	153	39.1	2.4
9.9	*30.2*	82.7	27.1	28.8	325	38.4	2.4
16.0	*47.5*	79.2	26.7	33.7	241	45.9	5.3
13.3	*33.2*	76.3	23.5	30.8	80	15.4	3.7
12.5	*38.6*	87.5	28.3	34.4	253	39.1	2.4
9.9	*30.2*	82.7	27.1	32.8	325	38.4	2.4
13.6	*43.7*	89.0	27.7	31.1	391	24.1	2.5
12.2	*38.6*	85.0	26.9	31.6	202	28.2	4.5
5.0	*14.3*	98.6	34.5	35.0	145	6.7	2.2
11.4	*24.7*	77.5	25.0	32.2	249	38.6	2.4
13.3	*40.9*	85.9	27.1	32.5	119	27.1	6.4
11.1	*34.4*	81.9	26.4	32.2	202	34.0	4.1
14.1	*31.7*	79.7	27.0	33.8	70	10.7	3.3
12.6	*36.1*	87.6	30.6	34.9	191	13.2	1.9
11.1	*27.7*	89.7	37.0	33.8	60	11.7	4.3
12.1	*34.9*	85.3	29.6	34.7	110	15.8	2.5
11.9	*35.9*	91.3	30.3	33.1	110	49.0	4.0
10.0	*29.6*	80.4	27.2	33.8	220	16.6	4.4
10.5	*31.7*	95.4	26.3	33.1	108	12.1	3.5
11.5	*45.0*	67.6	21.8	32.2	182	5.6	1.8
14.6	*32.9*	92.3	28.0	34.0	115	26.0	2.9
15.3	*47.2*	77.6	25.2	32.4	106	20.4	2.3
11.2	*29.4*	87.4	29.3	33.5	181	24.2	3.3

12.0	*33.3*	82.6	28.2	34.2	128	11.4	2.7
9.4	*30.7*	79.8	26.3	32.9	219	32.9	4.5
10.8	*37.9*	78.3	26.4	33.8	126	22.4	5.8
13.5	*40.0*	85.5	28.8	33.8	203	45.4	4.1
11.6	*20.7*	91.5	30.6	33.4	102	38.1	3.4

GR	RDWCV	RDWSD	PCT	MPV	PDW	CD4 (mm3	CD8 (mm3)
59.3	15.1	39.8	0.26	7.1	15.4	450	108
73.4	12.9	37.5	0.24	8.7	16.7	500	201
75.3	16.4	54.4	0.19	8.1	17.3	570	120
79.2	13.4	43.4	0.16	7.3	17.5	950	205
48.8	15.9	50.4	0.19	7.9	16.7	350	209
80.9	16.6	50.7	0.29	7.7	17.8	579	295
58.5	16.3	57.1	0.23	8.9	17.4	380	188
59.2	15.1	50.0	0.25	7.7	17.9	398	145
73.4	15.8	56.2	0.32	8.3	17.6	400	197
67.3	16.1	54.7	0.16	8.1	18.6	560	200
91.1	12.5	49.3	0.11	7.7	18.4	485	198
59.0	16.8	52.1	0.22	8.7	17.3	350	212
66.5	14.4	49.5	0.10	8.3	18.1	595	200
68.6	15.9	58.6	0.15	7.9	17.9	420	195
71.9	15.7	45.4	0.32	7.1	16.7	1158	388
65.3	13.9	43.1	0.17	7.7	18.3	589	289
62.4	14.4	45.9	0.18	8.1	17.3	380	235
88.0	13.0	41.3	0.15	7.9	17.4	590	255
58.1	13.5	42.9	0.21	7.3	17.6	329	198
59.4	13.2	46.1	0.23	8.5	16.5	468	190
55.8	16.1	49.7	0.36	8.1	16.9	400	188
55.0	15.3	49.1	0.23	7.7	16.5	888	175
82.1	14.5	49.5	0.20	8.1	17.3	970	205
44.1	14.4	49.7	0.19	9.8	16.3	300	187
33.4	12.4	40.3	0.22	7.9	16.2	298	154
35.1	12.4	42.8	0.16	9.4	16.7	440	143
21.1	14.3	40.1	0.21	8.3	17.6	457	167
29.1	12.5	41.7	0.20	7.7	17.0	320	185
39.1	13.6	43.4	0.17	7.7	16.6	400	200
71.8	12.7	39.8	0.11	8.5	17.2	453	187
50.5	13.5	46.2	0.16	7.9	15.5	550	204
82.8	13.3	44.1	0.11	7.9	17.8	520	265
51.3	13.4	43.2	0.19	8.5	15.4	430	300
63.5	13.4	44.6	0.15	7.3	16.8	990	310
69.0	13.0	42.7	0.22	7.9	16.6	454	198
79.7	13.6	46.8	0.20	8.7	16.7	476	198
69.2	13.3	43.4	0.26	7.5	16.2	540	188
57.6	13.3	44.6	0.19	7.7	17.8	542	187
67.6	12.9	41.8	0.05	8.3	17.6	958	167
61.8	13.4	43.2	0.17	7.9	16.6	400	206
62.0	13.8	42.0	0.16	8.7	16.8	490	158
61.9	11.8	38.7	0.15	7.3	16.8	560	360
86.0	13.9	44.3	0.19	6.9	17.2	570	401
84.9	13.4	47.0	0.17	8.7	16.1	500	405
81.7	14.4	49.1	0.08	7.7	17.8	990	595
47.0	14.9	54.4	0.09	8.5	17.6	460	301

79.0	17.0	54.7	0.17	7.7	17.1	459	295
84.4	14.5	46.1	0.10	9.1	17.5	557	309
92.6	1.2	38.4	0.15	8.1	17.4	420	310
71.1	12.6	41.5	0.10	8.5	18.3	438	325
77.3	13.6	42.2	0.13	7.9	17.4	600	210
72.5	12.9	45.1	0.12	6.9	18.6	530	427
85.9	13.3	43.9	0.17	7.3	17.5	560	450
39.2	14.0	41.4	0.12	8.1	18.1	998	305
54.5	13.6	39.3	0.22	8.3	17.6	460	396
76.7	14.3	51.1	0.06	8.6	18.7	398	265
59.6	14.9	47.7	0.21	7.5	17.0	498	296
45.5	13.9	47.5	0.11	7.7	17.8	998	550
72.5	12.9	42.3	0.14	8.1	16.9	544	350
51.6	12.8	39.7	0.19	7.3	17.6	455	300
78.4	13.8	42.9	0.08	7.7	17.8	520	301
76.4	13.4	37.5	0.18	8.1	17.4	490	299
66.6	15.7	46.8	0.10	7.5	18.3	495	305
66.1	13.5	42.9	0.16	8.5	17.7	420	286
68.5	13.7	43.5	0.06	8.4	20.2	480	305
77.7	13.5	42.6	0.20	8.1	18.6	421	298
61.8	13.5	43.8	0.14	8.1	18.1	430	285
78.5	14.4	48.4	0.06	9.0	18.5	1000	659
56.5	13.0	39.7	0.26	7.5	17.0	421	210
80.3	13.3	44.0	0.16	7.3	16.8	428	199
86.1	12.8	40.4	0.18	8.5	17.2	1420	300
59.2	13.8	44.0	0.15	7.1	17.3	460	309
64.3	14.7	42.3	0.18	8.7	16.6	390	299
63.2	14.6	51.6	0.21	9.1	18.6	431	240
64.7	13.6	45.0	0.19	8.1	17.3	339	198
57.2	13.1	44.4	0.21	8.7	17.2	228	197
64.0	16.5	49.4	0.25	8.3	16.9	520	296
79.7	22.2	78.3	0.25	8.5	15.4	450	209
61.1	13.0	41.9	0.16	8.5	17.2	431	298
69.3	15.9	48.4	0.14	7.5	16.3	985	500
70.3	12.3	40.0	0.17	7.1	16.7	477	285
64.6	13.7	44.6	0.19	7.7	17.9	265	175
61.1	13.7	43.6	0.17	8.3	17.6	380	265
73.4	16.9	55.3	0.17	8.1	18.1	440	199
74.2	13.1	42.0	0.21	7.9	17.3	320	216
52.2	13.3	44.9	0.18	7.9	16.8	280	187
55.4	12.6	42.7	0.25	8.7	17.2	420	217
19.8	17.7	59.9	0.05	8.9	19.3	890	450
63.4	12.3	38.5	0.17	6.9	18.6	425	195
49.5	12.8	39.0	0.16	8.5	18.8	365	186
67.6	14.2	45.5	0.17	7.9	17.8	405	294
28.5	15.3	56.0	0.23	8.5	16.5	208	155
67.4	14.1	47.3	0.06	8.0	19.0	880	600
51.8	13.1	43.5	0.18	7.9	17.3	409	198
71.0	16.7	49.6	0.27	7.9	17.2	389	165
81.9	15.7	45.4	0.32	8.1	16.7	1158	388

81.1	13.1	42.6	0.12	7.7	17.8	501	350
57.0	13.7	41.9	0.23	7.5	17.0	306	198
67.5	15.5	35.8	0.16	8.5	17.7	299	145
73.4	12.9	37.5	0.24	8.7	16.7	500	201
75.3	16.4	54.4	0.19	8.1	17.3	570	120
75.1	12.4	42.8	0.16	9.4	16.7	540	143
80.0	14.3	40.1	0.21	8.3	17.6	457	167
21.0	12.5	41.7	0.20	7.7	17.0	520	185
62.6	13.6	43.4	0.17	7.7	16.6	400	200
51.8	12.7	39.8	0.11	8.5	17.2	453	187
50.5	13.5	46.2	0.16	7.9	15.5	550	204
82.8	16.3	44.1	0.11	7.9	17.8	420	265
51.3	13.4	43.2	0.19	8.5	15.4	430	300
63.5	12.4	44.6	0.15	7.3	16.8	480	310
79.2	13.4	43.4	0.16	7.3	17.5	550	205
48.8	15.9	50.4	0.19	7.9	16.7	350	209
80.9	16.6	50.7	0.29	7.7	17.8	579	295
58.5	16.3	57.1	0.23	8.9	17.4	380	188
59.2	14.1	50.0	0.25	7.7	17.9	398	145
73.4	15.8	56.2	0.32	8.3	17.6	400	197
67.3	16.1	54.7	0.16	8.1	18.6	560	200
91.1	12.5	49.3	0.11	7.7	18.4	485	198
59.0	16.8	52.1	0.22	8.7	17.3	350	212
66.5	14.4	49.5	0.10	8.3	18.1	595	200
68.6	15.9	58.6	0.15	7.9	17.9	420	195
65.3	13.9	43.1	0.17	7.7	18.3	899	289
62.4	14.4	45.9	0.18	8.1	17.3	380	235
88.0	12.0	41.3	0.15	7.9	17.4	790	255
58.1	13.5	42.9	0.21	7.3	17.6	329	198
59.4	13.2	46.1	0.23	8.5	16.5	468	190
55.8	16.1	49.7	0.36	8.1	16.9	400	188
55.0	15.3	49.1	0.23	7.7	16.5	388	175
82.1	14.5	49.5	0.20	8.1	17.3	570	205
69.1	14.4	49.7	0.19	9.8	16.3	300	187
61.0	12.4	40.3	0.22	7.9	16.2	298	154
75.1	12.4	42.8	0.16	9.4	16.7	440	143
90.0	14.3	40.1	0.21	8.3	17.6	457	167
91.0	12.5	41.7	0.20	7.7	17.0	620	185
62.6	13.6	43.4	0.17	7.7	16.6	400	200
71.8	12.7	39.8	0.11	8.5	17.2	453	187
50.5	13.5	46.2	0.16	7.9	15.5	550	204
82.8	13.3	44.1	0.11	7.9	17.8	520	265
91.3	13.4	43.2	0.19	8.5	15.4	430	300
63.5	13.4	44.6	0.15	7.3	16.8	480	310
69.0	13.0	42.7	0.22	7.9	16.6	454	198
79.7	13.6	46.8	0.20	8.7	16.7	476	198
69.2	13.3	43.4	0.26	7.5	16.2	540	188
57.6	13.3	44.6	0.19	7.7	17.8	542	187
67.6	12.9	41.8	0.05	8.3	17.6	458	167
61.8	13.4	43.2	0.17	7.9	16.6	400	206

62.0	13.8	42.0	0.16	8.7	16.8	490	158
61.9	11.8	38.7	0.15	7.3	16.8	560	360
86.0	13.9	44.3	0.19	6.9	17.2	970	401
84.9	13.4	47.0	0.17	8.7	16.1	500	405
81.7	14.4	49.1	0.08	7.7	17.8	990	595
47.0	14.9	54.4	0.09	8.5	17.6	460	301
79.0	17.0	54.7	0.17	7.7	17.1	459	295
84.4	14.5	46.1	0.10	9.1	17.5	557	309
92.6	1.2	38.4	0.15	8.1	17.4	420	310
71.1	12.6	41.5	0.10	8.5	18.3	438	325
77.3	13.6	42.2	0.13	7.9	17.4	600	210
72.5	12.9	45.1	0.12	6.9	18.6	530	427
85.9	13.3	43.9	0.17	7.3	17.5	560	450
59.2	14.0	41.4	0.12	8.1	18.1	998	305
54.5	13.6	39.3	0.22	8.3	17.6	460	396
76.7	14.3	51.1	0.06	8.6	18.7	798	265
59.6	14.9	47.7	0.21	7.5	17.0	498	296
45.5	13.9	47.5	0.11	7.7	17.8	998	550
72.5	12.9	42.3	0.14	8.1	16.9	544	350
51.6	12.8	39.7	0.19	7.3	17.6	455	300
78.4	13.8	42.9	0.08	7.7	17.8	520	301
76.4	13.4	37.5	0.18	8.1	17.4	490	299
66.6	15.7	46.8	0.10	7.5	18.3	495	305
66.1	13.5	42.9	0.16	8.5	17.7	420	286
68.5	13.7	43.5	0.06	8.4	20.2	480	305
77.7	13.5	42.6	0.20	8.1	18.6	421	298
61.8	13.5	43.8	0.14	8.1	18.1	430	285
78.5	14.4	48.4	0.06	9.0	18.5	1000	659
56.5	13.0	39.7	0.26	7.5	17.0	421	210
80.3	13.3	44.0	0.16	7.3	16.8	428	199
86.1	12.8	40.4	0.18	8.5	17.2	520	300
59.2	13.8	44.0	0.15	7.1	17.3	660	309
64.3	14.7	42.3	0.18	8.7	16.6	390	299
63.2	14.6	51.6	0.21	9.1	18.6	831	240
64.7	13.6	45.0	0.19	8.1	17.3	339	198
57.2	13.1	44.4	0.21	8.7	17.2	228	197
64.0	16.5	49.4	0.25	8.3	16.9	720	296
79.7	22.2	78.3	0.25	8.5	15.4	450	209
61.1	13.0	41.9	0.16	8.5	17.2	431	298
69.3	15.9	48.4	0.14	7.5	16.3	985	500
70.3	12.3	40.0	0.17	7.1	16.7	477	285
73.4	15.6	52.5	0.15	6.5	19.7	389	167
64.6	13.7	44.6	0.19	7.7	17.9	265	175
61.1	13.7	43.6	0.17	8.3	17.6	380	265
73.4	16.9	55.3	0.17	8.1	18.1	440	199
74.2	13.1	42.0	0.21	7.9	17.3	320	216
52.2	13.3	44.9	0.18	7.9	16.8	280	187
55.4	12.6	42.7	0.25	8.7	17.2	720	217
69.8	17.7	59.9	0.05	8.9	19.3	890	450
63.4	12.3	38.5	0.17	6.9	18.6	425	195

49.5	13.8	39.0	0.16	8.5	18.8	365	186
67.6	14.2	45.5	0.17	7.9	17.8	605	294
58.5	15.3	56.0	0.23	8.5	16.5	208	155
67.4	14.1	47.3	0.06	8.0	19.0	880	600
51.8	13.1	43.5	0.18	7.9	17.3	409	198
71.0	16.7	49.6	0.27	7.9	17.2	389	165
81.9	15.7	45.4	0.32	8.1	16.7	458	388
81.1	14.1	42.6	0.12	7.7	17.8	501	350
57.0	13.7	41.9	0.23	7.5	17.0	306	198
67.5	12.5	35.8	0.16	8.5	17.7	299	145
76.7	14.3	51.1	0.06	8.6	18.7	398	265
59.6	14.9	47.7	0.21	7.5	17.0	498	296
45.5	13.9	47.5	0.11	7.7	17.8	998	550
72.5	12.9	42.3	0.14	8.1	16.9	544	350
51.6	12.8	39.7	0.19	7.3	17.6	455	300
78.4	13.8	42.9	0.08	7.7	17.8	520	301
76.4	13.4	37.5	0.18	8.1	17.4	490	299
66.6	15.7	46.8	0.10	7.5	18.3	495	305
66.1	13.5	42.9	0.16	8.5	17.7	420	286
68.5	13.7	43.5	0.06	8.4	20.2	480	305
77.7	13.5	42.6	0.20	8.1	18.6	421	298
61.8	13.5	43.8	0.14	8.1	18.1	430	285
86.1	12.8	40.4	0.18	8.5	17.2	520	300
59.2	13.8	44.0	0.15	7.1	17.3	460	309
64.3	14.7	42.3	0.18	8.7	16.6	390	299
63.2	14.6	51.6	0.21	9.1	18.6	431	240
64.7	13.6	45.0	0.19	8.1	17.3	339	198
57.2	13.1	44.4	0.21	8.7	17.2	228	197
64.0	16.5	49.4	0.25	8.3	16.9	520	296
79.7	22.2	78.3	0.25	8.5	15.4	450	209
61.1	13.0	41.9	0.16	8.5	17.2	431	298
69.3	15.9	48.4	0.14	7.5	16.3	985	500
70.3	12.3	40.0	0.17	7.1	16.7	477	285
64.6	13.7	44.6	0.19	7.7	17.9	265	175
61.1	13.7	43.6	0.17	8.3	17.6	380	265
73.4	16.9	55.3	0.17	8.1	18.1	440	199
74.2	13.1	42.0	0.21	7.9	17.3	320	216
52.2	13.3	44.9	0.18	7.9	16.8	280	187
58.5	15.3	56.0	0.23	8.5	16.5	208	155
67.4	14.1	47.3	0.06	8.0	19.0	880	600
51.8	13.1	43.5	0.18	7.9	17.3	409	198
71.0	16.7	49.6	0.27	7.9	17.2	389	165
81.9	15.7	45.4	0.32	8.1	16.7	458	388
81.1	13.1	42.6	0.12	7.7	17.8	501	350
57.0	13.7	41.9	0.23	7.5	17.0	306	198
67.5	15.5	35.8	0.16	8.5	17.7	299	145
73.4	12.9	37.5	0.24	8.7	16.7	500	201
75.3	16.4	54.4	0.19	8.1	17.3	570	120
79.2	13.4	43.4	0.16	7.3	17.5	550	205
48.8	15.9	50.4	0.19	7.9	16.7	1250	209

80.9	16.6	50.7	0.29	7.7	17.8	579	295
58.5	16.3	57.1	0.23	8.9	17.4	380	188
59.2	14.1	50.0	0.25	7.7	17.9	398	145
73.4	15.8	56.2	0.32	8.3	17.6	400	197
67.3	16.1	54.7	0.16	8.1	18.6	560	200
91.1	12.5	49.3	0.11	7.7	18.4	485	198
59.0	16.8	52.1	0.22	8.7	17.3	350	212
66.5	14.4	49.5	0.10	8.3	18.1	595	200
59.2	14.0	41.4	0.12	8.1	18.1	998	305
54.5	13.6	39.3	0.22	8.3	17.6	460	396
76.7	14.3	51.1	0.06	8.6	18.7	398	265
59.6	14.9	47.7	0.21	7.5	17.0	498	296
45.5	13.9	47.5	0.11	7.7	17.8	998	550
72.5	12.9	42.3	0.14	8.1	16.9	544	350
51.6	12.8	39.7	0.19	7.3	17.6	455	300
78.4	13.8	42.9	0.08	7.7	17.8	520	301
51.8	13.1	43.5	0.18	7.9	17.3	409	198
71.0	16.7	49.6	0.27	7.9	17.2	389	165
81.9	15.7	45.4	0.32	8.1	16.7	1258	388
81.1	13.1	42.6	0.12	7.7	17.8	501	350
57.0	13.7	41.9	0.23	7.5	17.0	306	198
67.5	15.5	35.8	0.16	8.5	17.7	299	145
73.4	12.9	37.5	0.24	8.7	16.7	500	201
75.3	16.4	54.4	0.19	8.1	17.3	570	120
79.2	13.4	43.4	0.16	7.3	17.5	550	205
48.8	15.9	50.4	0.19	7.9	16.7	350	209
80.9	16.6	50.7	0.29	7.7	17.8	1250	295
58.5	16.3	57.1	0.23	8.9	17.4	380	188
59.2	14.1	50.0	0.25	7.7	17.9	398	145
48.8	15.9	50.4	0.19	7.9	16.7	350	209
80.9	16.6	50.7	0.29	7.7	17.8	779	295
58.5	16.3	57.1	0.23	8.9	17.4	380	188
59.2	15.1	50.0	0.25	7.7	17.9	398	145
73.4	15.8	56.2	0.32	8.3	17.6	400	197
67.3	16.1	54.7	0.16	8.1	18.6	560	200
91.1	12.5	49.3	0.11	7.7	18.4	485	198
59.0	16.8	52.1	0.22	8.7	17.3	900	212
66.5	14.4	49.5	0.10	8.3	18.1	595	200
61.9	11.8	38.7	0.15	7.3	16.8	560	360
86.0	13.9	44.3	0.19	6.9	17.2	970	401
84.9	13.4	47.0	0.17	8.7	16.1	500	405
86.0	13.9	44.3	0.21	6.9	19.2	978	501
81.7	14.4	49.1	0.08	7.7	17.8	990	595
47.0	14.9	54.4	0.09	8.5	17.6	460	301
79.0	17.0	54.7	0.17	7.7	17.1	459	295
84.4	14.5	46.1	0.10	9.1	17.5	557	309
92.6	1.2	38.4	0.15	8.1	17.4	420	310
71.1	12.6	41.5	0.10	8.5	18.3	1038	325
77.3	13.6	42.2	0.13	7.9	17.4	600	210
72.5	12.9	45.1	0.12	6.9	18.6	930	427

85.9	13.3	43.9	0.17	7.3	17.5	560	450
62.6	13.6	43.4	0.17	7.7	16.6	400	200
71.8	12.7	39.8	0.11	8.5	17.2	453	187
50.5	13.5	46.2	0.16	7.9	15.5	550	204
58.5	15.3	56.0	0.23	8.5	16.5	1008	155

Germe isole	PENG	AMX	AMC (30µg)	CAZ (30µg)
salmonella paratyphi A		I	R	S
E.Coli		R	S	R
shigella spp		R	S	R
E.Coli		R	S	R
salmonella paratyphi A		S	R	S
E.Coli		R	S	R
proteus mirabilis		S	R	R
staphyloccus aureus	S	R	S	S
Salmonella arizonae		S	R	I
enterobacter agglomerans		R	S	R
staphyloccus aureus	S	S	R	S
E.Coli		R	S	R
KLEBSIELLA pneumonae		S	I	S
salmonella spp		R	R	S
Pseudomonas aurogenosa		I	S	R
E.Coli		R	S	R
Proteus morganii		S	S	S
E.Coli		S	R	R
shigella spp		R	S	R
Serratia odorifera biogroupe 1		R	R	R
KLEBSIELLA oxytoca		S	S	S
Serratia odorifera biogroupe 1		*R*	R	R
E.Coli		R	R	R
SALMONELLA spp		S	S	S
E.Coli		S	R	S
Proteus morganii		R	S	R
staphyloccus aureus	R	S	R	S
Serratia liquefaciens		*R*	R	R
salmonella paratyphi A		R	S	S
proteus mirabilis		R	I	R
Serratia odorifera biogroupe 1		R	R	R
Serratia odorifera biogroupe 1		R	R	R
E.Coli		R	S	S
SERRATIA odorifera biogroupe 1		S	S	R
E.Coli		S	R	R
enterobacter cloacae		R	S	S
Klebsiella pneumonae		S	R	I
E.Coli		S	R	S
pseudomonas aurogenosa		R	S	R
Serratia odorifera biogroupe 1		S	R	R
citrobacter spp		S	I	S
shigella spp		R	S	R
Proteus morganii		S	R	S
E.COLI		S	S	S
staphyloccus aureus	R	S	R	R
Salmonella arizonae		R	S	R

enterobacter cloacae		R	S	R
Serratia odorifera biogroupe 1		S	R	S
E.Coli		R	S	R
enterobacter agglomerans		R	R	S
Serratia liquefaciens		S	R	R
staphyloccus aureus		R	S	R
proteus mirabilis		S	R	S
Salmonella arizonae		S	R	R
PSEUDOMONAS fluorescent		S	S	S
Proteus morganii		S	S	S
salmonella spp		R	R	R
Pseudomonas aurogenosa		R	S	R
shigella spp		I	R	S
E.Coli		R	S	R
Salmonella paratyphi A		R	S	R
enterobacter agglomerans		S	R	R
SALMONELLA spp		R	S	R
E.Coli		R	S	S
salmonella paratyphi A		S	R	R
Proteus morganii		R	S	R
klebsiella oxytoca		S	R	R
Salmonella arizonae		S	I	S
E.Coli		R	R	R
staphyloccus aureus	R	S	R	R
Serratia odorifera biogroupe 1		S	R	R
Serratia odorifera biogroupe 1		R	R	R
E.Coli		S	S	S
Serratia odorifera biogroupe 1		S	R	R
Serratia liquefaciens		S	R	R
pseudomonas aeruginosa		R	S	R
E.Coli		R	I	R
proteus mirabilis		R	R	S
E.Coli		I	S	R
proteus mirabilis		R	S	R
shigella spp		R	R	R
staphyloccus aureus	R	S	R	R
E.Coli		S	I	S
Serratia odorifera biogroupe 1		S	R	R
Serratia odorifera biogroupe 1		R	R	R
salmonella paratyphi A		R	S	S
staphyloccus aureus	R	S	R	R
Salmonella arizonae		S	R	R
Proteus morganii		R	S	R
Serratia odorifera biogroupe 1		S	R	R
Klebsiella pneumonae		S	S	S
E.coli		R	S	R
PSEUDOMONAS aeruginosa		S	S	R
Serratia odorifera biogroupe 1		R	R	R
E.Coli		R	R	S
KLEBSIELLA oxytoca		S	R	S

Enterobacter agglomerans		R	S	R
Klebsiella oxytoca		S	S	S
E.coli		S	R	R
Enterobacter agglomerans		R	R	R
Salmonella paratyphi A		S	R	S
STAPHYLOCCOCUS aureus	S	S	S	R
PSEUDOMONAS aurogenosa		S	R	S
PSEUDOMONAS aeruginosa		R	S	R
E.COLI		S	S	S
SERRATIA odorifera biogroupe 1		S	R	R
Klebsiella pneumonae		S	R	S
Serratia odorifera biogroupe 1		R	S	S
Enterobacter agglomerans		S	R	R
PROTEUS mirabilis		S	R	S
E.Coli		S	R	R
Pseudomonas fluorescent		R	S	R
E.Coli		S	R	S
proteus mirabilis		S	R	S
Enterobacter cloacae		R	S	R
Salmonella arizonae		S	S	S
E.Coli		S	R	R
staphyloccus aureus	R	S	S	S
E.Coli		S	R	R
Klebsiella pneumonae		R	R	S
salmonella paratyphi A		R	S	R
Pseudomonas aurogenosa		I	R	R
E.Coli		R	S	S
salmonella spp		R	R	R
E.Coli		S	R	R
Proteus morganii		S	S	S
staphyloccus aureus	S	R	R	S
E.Coli	R	I	R	R
Serratia odorifera biogroupe 1		S	R	S
proteus mirabilis		S	I	S
staphyloccus aureus	R	S	S	R
klebsiella oxytoca		S	R	R
citrobacter spp		R	S	S
proteus mirabilis		S	R	I
Serratia liquefaciens		*R*	S	R
E.Coli		R	R	R
pseudomonas aeruginosa		R	S	S
Serratia odorifera biogroupe 1		R	S	S
Serratia odorifera biogroupe 1		S	R	S
Klebsiella pneumonae		S	R	R
Serratia odorifera biogroupe 1		S	R	S
Klebsiella pneumonae		I	R	R
Serratia odorifera biogroupe 1		S	R	S
enterobacter agglomerans		S	I	S
E.Coli		S	S	S
citrobacter spp		R	R	R

Serratia odorifera biogroupe 1		R	I	R
proteus mirabilis		R	R	S
shigella spp		S	R	R
salmonella paratyphi A		S	R	S
E.Coli		R	S	R
staphyloccus aureus	S	S	R	R
E.Coli		R	R	R
staphyloccus aureus	R	R	R	R
Serratia odorifera biogroupe 1		R	S	R
Enterobacter cloacae		S	R	S
enterobacter agglomerans		R	S	S
Serratia liquefaciens		I	R	S
Enterobacter cloacae		R	R	S
E.Coli		S	R	S
Salmonella arizonae		R	S	R
Salmonella arizonae		S	R	S
enterobacter cloacae		S	R	R
Serratia odorifera biogroupe 1		R	R	R
Pseudomonas aurogenosa		I	R	R
proteus mirabilis		R	S	R
salmonella paratyphi A		S	R	R
proteus mirabilis		R	S	R
E.Coli		R	S	S
SALMONELLA arizonae		S	R	S
staphyloccus aureus	R	R	S	R
Proteus morganii		R	R	R
klebsiella oxytoca		R	R	R
pseudomonas aeruginosa		S	R	R
Serratia spp		S	S	S
Enterobacter cloacae		R	R	S
Serratia odorifera biogroupe 1		S	S	S
SERRATIA odorifera biogroupe 1		R	S	R
Enterobacter cloacae		S	R	I
enterobacter agglomerans		S	S	S
E.Coli		S	R	S
staphyloccus aureus	R	R	R	S
Salmonella arizonae		S	R	I
enterobacter agglomerans		S	R	S
staphyloccus aureus		R	S	R
staphyloccus aureus	R	R	R	R
pseudomonas aeruginosa		S	I	I
E.Coli		I	R	R
Proteus morganii		S	S	R
SALMONELLA paratyphi A		S	S	R
Klebsiella pneumonae		R	R	S
E.Coli		R	R	S
shigella spp		S	R	S
proteus mirabilis		R	S	R
E.Coli		R	S	S
Klebsiella pneumonae		R	S	S

proteus mirabilis		R	S	R
E.COLI		S	S	R
E.Coli		S	R	S
proteus mirabilis		R	R	R
staphyloccus aureus	I	R	R	S
E.Coli		S	R	S
SHIGELLA spp		S	S	S
E.Coli		R	S	S
pseudomonas aeruginosa	R	S	R	R
salmonella spp		I	R	R
E.Coli		S	R	S
Proteus morganii		S	I	S
klebsiella oxytoca		S	R	S
Klebsiella pneumonae		S	R	S
proteuse mirabilis		R	S	R
Serratia spp		R	R	R
enterobacter agglomerans		R	R	S
Proteus morganii		S	R	S
SHIGELLA spp		S	S	R
shigella spp		R	S	S
proteus mirabilis		S	R	S
E.Coli		R	S	S
Klebsiella pneumonae		S	R	R
Pseudomonas fluorescent		R	S	R
Proteus morganii		S	R	S
SALMONELLA spp		R	S	R
shigella spp		I	R	R
E.Coli		S	R	S
Klebsiella pneumonae		R	S	R
Salmonella arizonae		R	S	R
enterobacter agglomerans		R	I	R
SALMONELLA spp		R	R	S
E.Coli		I	S	R
Klebsiella pneumonae		R	S	R
E.Coli		R	R	S
Proteus morganii		S	R	S
shigella spp		R	S	R
Klebsiella pneumonae		S	R	R
E.Coli		R	S	R
PROTEUS mirabilis		S	S	S
Serratia odorifera biogroupe 1		S	R	R
proteus mirabilis		R	S	S
Enterobacter cloacae		S	R	S
Enterobacter cloacae		R	S	R
salmonella spp		S	R	R
E.Coli		R	R	R
shigella spp		S	R	S
E.Coli		R	S	R
salmonella spp		R	S	R
E.Coli		S	R	R

Proteus morganii		R	R	R
staphyloccus aureus	R	R	S	R
enterobacter agglomerans		S	R	S
Pseudomonas fluorescent		S	R	R
PROTEUS morganii		S	S	S
SALMONELLA spp		S	R	S
shigella spp		R	R	R
PROTEUS mirabilis		R	S	S
E.Coli		R	S	S
Salmonella arizonae		S	R	S
STAPHYLOCCOCUS aureus	R	S	S	R
proteus mirabilis		R	R	S
klebsiella oxytoca		S	R	R
E.Coli		S	R	S
Proteus morganii		R	S	R
klebsiella oxytoca		S	R	R
Serratia odorifera biogroupe 1		R	R	R
E.Coli		R	S	S
SHIGELLA spp		S	R	S
Proteus morganii		R	R	R
E.Coli		S	R	R
E.Coli		R	S	R
ENTEROBACTER cloacae		S	R	S
Klebsiella pneumonae		S	R	R
E.Coli		R	S	S
STAPHYLOCOCCUS aureus	S	S	R	S
pseudomonas aeruginosa		S	R	S
salmonella spp		R	S	R
staphyloccus aureus	S	S	R	S
citrobacter spp		S	R	R
KLEBSIELLA pneumonae		S	R	S
shigella spp		S	R	R
shigella spp		S	R	R
proteus mirabilis		R	R	S
E.COLI		R	R	S
Klebsiella pneumonae		S	S	R
pseudomonas aeruginosa		R	R	S
E.COLI		S	S	R
Enterobacter cloacae		S	R	S
enterobacter agglomerans		R	S	S
Serratia liquefaciens		S	S	R
PROTEUS mirabilis		R	S	R
Klebsiella pneumonae		R	R	R
E.Coli		R	S	S
E.COLI		S	S	S
pseudomonas aeruginosa		S	R	S
Serratia spp		S	R	R
ENTEROBACTER cloacae		S	R	S
proteuse mirabilis		R	S	S
SERRATIA spp		R	R	R

PSEUDOMONAS fluorescent		S	S	R
PROTEUS MORGANII		S	S	S
SAL MONLELLA SPP		S	S	S
shigella spp		S	S	S
STAP AUREUS	R	S	S	S

CIP(5μg)	LEV (5μg)	CHL (μg)	CDA (μg)	ERY(μg)	SXT (μg)	COT (μg)	DOX (μg)
R	S	R	R	R	R	S	R
S	R	R	R	S	R	R	R
S	R	R	R	S	R	S	R
R	S	R	R	R	R	R	R
R	S	R	R	S	S	I	S
R	S	R	R	R	S	R	R
S	R	S	R	R	R	S	R
S	R	S	R	S	R	S	R
R	R	I	R	R	R	R	R
S	R	R	S	R	S	R	R
R	R	S	R	R	R	R	R
S	I	I	S	R	R	I	S
S	R	S	S	S	S	R	S
R	S	S	R	S	R	R	S
R	I	R	S	R	R	S	R
S	S	R	S	R	S	I	S
R	R	R	S	R	R	R	R
S	S	R	R	S	S	S	R
S	S	R	R	S	S	R	R
S	S	S	S	R	R	S	R
S	S	S	S	R	S	S	S
S	S	R	S	S	R	S	R
S	S	S	I	R	I	S	I
S	R	S	S	R	S	R	S
R	R	S	R	R	R	R	R
S	R	R	S	R	S	R	R
R	R	S	R	R	R	R	R
S	S	S	S	S	R	S	S
S	S	R	S	S	S	R	S
R	S	R	S	R	S	R	R
S	S	S	S	S	S	R	S
S	S	S	S	S	R	S	R
R	S	S	S	R	R	R	S
S	S	S	S	R	S	S	S
S	R	S	R	R	R	S	R
S	R	S	R	S	R	S	R
R	R	I	R	R	R	R	R
R	R	S	R	R	R	R	S
S	R	R	S	I	R	S	R
S	S	S	S	R	S	S	R
S	I	S	I	S	S	R	R
S	R	R	S	R	S	R	R
R	R	S	R	R	R	R	R
S	R	S	S	I	I	S	S
R	S	R	R	R	R	R	R
R	R	R	R	R	S	R	R

I	I	S	S	I	S	S	R
R	R	S	S	R	R	S	S
S	S	S	R	R	S	R	S
S	R	S	S	R	S	R	S
R	R	S	S	R	S	R	R
I	I	S	S	I	S	S	R
R	R	S	S	R	R	S	S
S	R	S	R	R	R	S	R
R	R	S	R	R	S	S	S
S	S	S	S	S	S	S	S
R	R	R	R	R	R	R	R
S	R	R	R	S	R	S	R
R	S	R	R	R	R	S	R
S	R	R	R	S	R	R	R
R	S	R	R	R	R	R	R
S	S	R	S	R	R	S	I
R	S	R	R	R	S	R	S
S	R	R	R	I	I	S	S
R	S	R	R	R	R	R	R
R	R	R	R	R	S	R	R
S	S	S	S	R	S	S	R
S	I	S	I	S	S		R
R	R	R	R	R	R	R	R
S	S	S	R	R	S	S	S
S	S	S	S	S	R	S	R
S	S	R	S	R	R	R	S
S	S	R	S	S	R	S	R
R	R	S	S	S	S	R	S
S	S	S	S	R	R	S	R
S	I	I	S	R	R	I	S
S	R	S	S	R	S	R	R
R	S	S	R	S	R	R	S
R	I	R	S	R	R	S	R
R	R	R	S	S	R	R	R
S	S	R	R	R	S	S	R
S	S	S	S	R	S	S	R
S	I	S	I	S	S	R	R
R	S	S	S	S	R	R	S
R	S	S	S	R	S	S	S
S	S	R	R	R	S	S	R
R	S	R	R	R	R	S	S
R	S	R	R	S	R	R	S
R	R	R	R	R	R	R	R
S	R	S	S	S	I	S	R
R	R	I	R	R	S	R	S
R	S	R	R	R	R	S	R
S	S	S	S	R	S	S	S
R	S	S	S	R	R	S	R
S	R	R	R	S	S	S	R
R	S	R	S	S	S	S	R

S	S	R	S	R	S	I	S
R	R	R	S	R	R	R	R
S	S	R	R	S	S	S	R
R	R	R	R	R	R	R	R
R	R	R	S	S	R	R	R
S	R	S	R	S	S	S	R
S	S	S	S	S	R	S	R
R	S	R	S	R	S	S	R
S	R	R	S	S	R	S	S
S	S	R	S	S	S	R	R
R	R	S	S	S	R	S	S
S	I	I	R	S	S	I	S
S	R	R	R	S	R	R	R
S	S	S	S	R	S	S	S
S	S	S	S	S	R	S	R
R	R	S	R	R	S	R	S
R	R	S	R	R	R	S	R
S	S	S	R	R	R	S	S
S	S	R	S	R	S	I	S
R	R	R	S	R	R	R	R
S	S	R	R	S	S	S	R
S	S	R	S	R	S	I	S
R	S	S	R	R	S	R	R
R	R	R	R	R	R	R	R
S	S	R	R	S	S	S	S
S	R	R	R	R	R	R	R
R	S	S	S	S	S	S	S
R	I	R	R	R	R	R	I
S	S	R	S	S	S	I	S
R	R	R	S	S	S	R	S
S	R	S	S	R	S	S	R
R	S	R	R	S	R	S	R
R	R	R	R	R	S	R	R
R	S	S	R	R	R	S	R
S	R	S	R	R	S	S	S
S	R	S	R	R	R	S	R
S	R	S	R	S	R	S	R
R	R	I	R	R	R	R	R
R	I	S	R	S	I	R	S
R	R	R	R	R	S	R	R
S	R	R	S	S	S	R	R
R	S	R	S	S	S	R	I
R	R	R	S	S	R	R	R
S	R	S	R	R	R	S	R
R	S	R	S	S	S	R	R
R	S	R	R	S	R	S	R
R	R	R	R	R	S	R	R
R	S	S	R	R	R	S	R
R	R	S	R	S	R	S	S
S	S	R	R	I	I	S	R

R	R	R	R	R	R	R	R
R	R	R	R	I	R	R	S
R	R	R	R	R	S	R	R
R	S	R	S	S	S	R	S
S	S	R	S	S	R	S	R
R	S	S	R	R	R	R	S
R	R	R	R	R	R	R	R
R	R	R	R	R	R	I	R
R	R	S	R	S	R	R	R
R	R	R	R	S	R	R	R
S	S	R	S	S	S	R	R
S	R	R	S	R	S	R	R
R	S	S	S	R	S	R	S
R	S	S	S	S	R	R	S
S	R	S	S	R	R	R	S
R	S	R	S	R	S	S	R
R	S	R	R	R	R	S	R
R	R	R	R	R	R	I	R
R	R	S	R	S	R	R	R
R	S	R	R	R	R	R	R
S	S	R	S	R	R	S	I
R	S	R	R	R	S	R	R
S	R	R	R	I	I	S	S
R	S	S	S	R	S	S	R
R	R	R	R	R	S	R	R
R	R	S	R	R	R	R	S
R	I	R	R	R	R	R	I
S	S	R	S	S	S	I	S
R	R	R	S	S	S	R	S
S	R	S	S	R	S	S	R
S	R	S	S	S	R	S	R
S	R	R	S	S	R	R	R
R	R	R	R	R	R	S	R
R	S	S	R	R	S	S	S
R	R	R	R	S	R	S	R
R	R	S	R	R	R	I	R
R	R	I	R	R	R	R	R
R	R	S	R	R	R	R	S
S	R	R	S	I	R	S	R
I	R	R	R	R	R	R	R
R	S	R	S	S	S	R	R
S	R	R	R	R	R	R	S
R	R	S	S	S	S	R	S
S	R	S	S	R	R	S	R
S	R	R	S	S	R	S	R
R	S	S	S	R	S	R	S
R	S	S	S	S	R	R	S
R	S	S	S	S	R	S	R
S	R	S	S	S	R	S	R
R	S	R	S	R	S	S	R

S	I	S	S	S	R	R	S
S	R	S	R	S	S	S	R
S	S	S	S	S	R	S	S
R	R	R	R	R	R	R	R
R	S	S	S	R	S	R	S
R	S	S	S	S	R	R	S
R	R	S	S	R	S	S	R
R	S	R	S	S	R	S	R
R	R	R	R	S	R	R	R
R	S	R	R	S	R	S	R
R	R	R	R	R	S	R	R
R	S	S	R	R	R	S	R
R	S	R	S	S	R	S	R
R	S	R	S	R	S	R	R
S	R	S	S	R	S	S	R
R	R	S	R	R	R	R	S
S	S	I	S	S	R	S	R
R	R	R	R	R	S	R	R
S	S	S	R	S	S	S	S
R	S	S	S	R	R	R	S
R	R	S	S	S	R	S	S
S	I	I	R	S	S	I	S
R	R	S	R	R	R	R	R
R	R	R	R	R	R	S	R
R	R	S	S	S	R	S	S
R	S	R	S	R	S	I	R
R	R	S	R	S	R	R	R
R	R	S	S	S	R	S	S
S	R	S	R	S	S	R	S
S	I	I	S	R	R	I	S
S	R	S	S	R	S	R	R
R	S	S	R	S	R	R	S
R	I	R	S	R	R	S	R
S	R	S	R	R	S	R	S
S	S	R	R	S	S	R	S
R	R	R	R	R	S	R	R
S	S	S	R	S	R	S	S
S	R	R	R	S	R	S	R
R	R	S	R	R	R	R	R
S	R	S	R	S	S	S	S
S	S	I	R	I	S	R	S
R	I	I	R	I	S	R	R
R	S	R	S	S	S	R	S
S	S	R	S	S	R	S	R
R	S	S	R	R	R	R	S
R	R	R	R	R	R	R	R
R	R	R	R	R	S	R	R
S	S	S	R	S	R	S	S
S	I	I	S	S	S	R	R
SS	R	S	R	R	R	R	S

S	R	R	R	S	R	R	R
S	R	R	S	R	S	R	R
R	S	R	R	R	S	S	R
R	R	S	R	S	R	R	S
S	S	S	S	S	S	S	S
R	S	R	R	S	R	S	R
R	R	R	R	R	S	R	R
S	R	R	S	S	S	R	R
R	S	R	S	S	S	R	I
R	R	R	S	S	R	R	R
S	S	S	R	S	R	S	R
R	R	R	R	I	R	R	S
R	R	R	R	R	S	R	R
R	S	R	S	S	S	R	S
S	S	R	S	S	R	S	R
R	S	S	R	R	R	R	S
	S	S	S	R	S	R	S
S	I	S	S	R	R	S	R
R	R	S	R	S	R	S	S
R	R	R	R	R	R	R	R
S	R	R	R	S	R	S	R
R	R	S	R	R	R	R	R
S	R	S	R	S	R	S	S
S	S	I	R	I	S	R	S
R	I	I	R	I	S	R	R
S	R	S	R	S	R	S	S
R	S	R	R	S	R	R	S
R	R	S	R	R	S	R	R
R	R	R	S	S	R	R	R
S	R	S	R	R	R	S	R
R	S	R	S	R	S	R	R
S	S	S	R	R	S	S	S
S	S	S	S	S	R	S	R
R	R	R	S	S	R	S	S
R	I	S	R	R	S	R	I
R	S	S	R	R	S	R	S
R	S	R	R	R	R	R	S
S	R	S	S	R	S	S	R
R	R	R	R	S	R	R	R
S	S	S	S	S	R	S	S
R	S	S	S	R	R	S	R
R	R	S	S	S	S	S	S
R	R	R	R	R	S	R	R
S	R	S	S	S	S	R	R
R	S	S	S	S	S	R	I
R	R	R	S	S	R	R	R
S	R	S	R	R	R	S	R
S	R	S	R	S	S	S	R
R	R	R	S	S	S	R	S
S	R	R	R	R	R	R	R

S	S	S	S	S	S	S	I
R	S	S	R	S	S	I	S
S	S	R	S	S	S	S	S
S	S	S	S	S	S	S	S
R	S	R	R	R	S	R	S

AKI (0µg)	MDR	QN	AT	GC	ATA	MLX	NEM	SUR
S	**1**	128	**256**	**128**	**256**	**256**	**128**	**256**
R	**1**	128	**256**	**128**	**256**	**256**	**128**	**256**
R	**1**	32	64	32	32	64	32	256
R	**0**	64	64	256	128	128	128	64
S	**0**	128	**256**	**128**	**256**	**256**	**128**	**256**
R	**1**	32	64	256	128	128	128	256
R	**1**	128	**256**	**128**	**256**	**256**	**128**	**256**
R	**1**	64	64	256	128	128	128	64
R	**1**	128	**256**	**128**	**256**	**256**	**128**	**256**
R	**0**	32	128	64	128	128	128	64
R	**1**	128	**256**	**128**	**256**	**256**	**128**	**256**
R	**0**	64	64	256	128	128	128	256
S	**0**	32	64	256	128	128	128	64
R	**1**	128	**256**	**128**	**256**	**256**	**128**	**256**
R	**1**	64	256	256	128	128	128	256
R	**0**	128	**256**	**128**	**256**	**256**	**128**	**256**
R	**1**	128	**256**	**128**	**256**	**128**	**128**	**256**
R	**0**	32	64	256	128	128	128	128
R	**0**	128	**256**	**128**	**256**	**256**	**128**	**256**
R	**0**	128	**256**	**128**	**256**	**128**	**128**	**256**
S	**0**	32	256	256	128	128	128	128
R	**1**	128	**256**	**128**	**256**	**256**	**128**	**256**
R	**1**	128	64	256	128	128	128	256
S	**0**	32	64	256	128	128	128	64
R	**1**	256	**256**	**128**	**256**	**128**	**128**	**256**
R	**1**	128	**256**	**256**	**256**	**256**	**128**	**256**
R	**0**	64	256	256	128	128	128	256
R	**0**	128	**256**	**128**	**256**	**256**	**128**	**256**
R	**0**	128	**256**	**128**	**256**	**128**	**128**	**256**
R	**0**	128	**256**	**128**	**256**	**256**	**128**	**256**
R	**1**	128	**256**	**128**	**256**	**256**	**128**	**256**
R	**0**	128	**256**	**128**	**256**	**256**	**128**	**256**
R	**0**	128	**256**	**128**	**256**	**256**	**256**	**256**
S	**1**	32	64	**128**	128	128	128	128
R	**1**	64	256	**128**	128	128	128	128
R	**1**	32	64	**128**	128	128	128	256
R	**0**	256	**256**	**128**	**256**	**128**	**128**	**256**
R	**0**	128	**256**	**128**	**256**	**256**	**128**	**256**
R	**1**	32	128	**128**	128	128	128	64
R	**0**	128	**256**	**128**	**256**	**128**	**128**	**256**
R	**0**	128	**256**	**128**	**256**	**256**	**128**	**256**
R	**0**	128	**256**	**128**	**256**	**256**	**128**	**256**
R	**0**	64	64	**128**	128	128	128	128
R	**1**	64	64	**128**	128	128	128	64
R	**0**	64	64	**128**	128	128	128	128
R	**0**	128	**256**	**128**	**256**	**256**	**128**	**256**

R	**1**	32	128	**128**	128	128	128	64
R	**0**	128	64	**128**	128	128	128	256
R	**0**	256	**256**	**128**	**256**	**128**	**128**	**256**
R	**0**	128	**256**	**128**	**256**	**256**	**128**	**256**
R	**0**	32	64	**128**	128	128	128	128
R	**0**	256	**256**	**128**	**256**	**128**	**128**	**256**
R	**0**	128	**256**	**128**	**256**	**256**	**128**	**256**
R	**1**	32	256	**128**	128	128	128	64
R	**0**	64	64	**128**	128	128	128	128
R	**0**	128	**256**	**128**	**256**	**256**	**128**	**256**
R	**0**	32	64	**128**	128	128	128	256
R	**1**	64	64	**128**	128	128	128	64
R	**0**	32	256	**128**	128	128	128	64
R	**0**	256	**256**	**128**	**256**	**128**	**128**	**256**
R	**0**	128	**256**	**128**	**256**	**256**	**128**	**256**
R	**1**	32	64	**128**	128	128	128	128
R	**0**	128	**256**	**128**	**256**	**256**	**128**	**256**
R	**0**	128	**256**	**128**	**256**	**256**	**128**	**256**
R	**0**	128	**256**	**128**	**256**	**256**	**128**	**256**
R	**1**	128	**256**	**128**	**256**	**256**	**128**	**256**
R	**0**	128	**256**	**128**	**256**	**256**	**128**	**256**
R	**1**	128	**256**	**128**	**256**	**256**	**128**	**256**
R	**1**	256	**256**	**128**	**256**	**128**	**128**	**256**
R	**1**	128	**256**	**128**	**256**	**256**	**128**	**256**
R	**0**	64	64	**128**	128	128	128	64
R	**0**	256	**256**	**128**	**256**	**128**	**128**	**256**
R	**0**	128	**256**	**128**	**256**	**256**	**128**	**256**
R	**0**	32	128	**128**	128	128	128	256
R	**0**	128	**256**	**128**	**256**	**256**	**128**	**256**
R	**1**	128	**256**	**128**	**256**	**256**	**128**	**256**
R	**1**	256	**256**	**128**	**128**	**256**	**128**	**256**
R	**0**	128	**256**	**128**	**256**	**256**	**128**	**256**
R	**0**	128	**256**	**128**	**256**	**256**	**128**	**256**
R	**0**	64	64	**128**	128	128	128	64
R	**0**	128	**256**	**128**	**256**	**256**	**128**	**256**
R	**0**	128	**256**	**128**	**256**	**256**	**128**	**256**
R	**0**	256	**256**	**128**	**128**	**256**	**128**	**256**
R	**0**	128	**256**	**128**	**256**	**256**	**128**	**256**
R	**1**	128	64	**128**	128	128	128	128
R	**0**	256	**256**	**128**	**256**	**128**	**128**	**256**
R	**0**	128	**256**	**128**	**256**	**256**	**128**	**256**
R	**1**	32	128	**128**	128	128	128	64
R	**0**	128	**256**	**128**	**256**	**256**	**128**	**256**
R	**0**	128	**256**	**128**	**256**	**256**	**128**	**256**
R	**1**	128	**256**	**128**	**256**	**256**	**128**	**256**
R	**1**	128	**256**	**128**	**256**	**256**	**128**	**256**
S	**1**	128	**256**	**128**	**256**	**256**	**128**	**256**
R	**1**	128	**256**	**128**	**256**	**256**	**128**	**256**
R	**0**	128	**256**	**128**	**256**	**256**	**128**	**256**
R	**0**	128	**256**	**128**	**256**	**256**	**128**	**256**

R	0	128	256	128	256	256	128	256
R	0	128	256	128	256	256	128	256
R	0	128	256	128	256	256	128	256
R	0	128	256	128	256	256	128	256
R	0	128	256	128	256	256	128	256
R	1	32	64	128	128	128	128	64
R	1	64	128	128	128	128	128	256
R	1	32	64	128	128	64	128	256
R	0	32	64	128	128	128	128	64
R	0	64	64	128	128	128	128	128
R	0	256	256	128	256	128	128	256
R	0	128	256	128	256	256	128	256
R	0	32	64	128	128	128	128	64
R	0	64	64	128	128	128	128	128
R	1	64	256	128	128	128	128	64
R	0	128	256	128	256	256	128	256
R	0	32	64	128	128	128	128	64
R	0	64	256	128	128	128	128	256
R	0	256	256	128	256	128	128	256
R	0	128	256	128	256	256	128	256
R	0	32	64	128	128	128	128	64
R	0	64	128	128	128	128	128	128
R	0	128	256	128	256	256	128	256
R	0	256	256	128	256	128	128	256
R	0	128	256	128	256	256	128	256
R	0	32	64	128	128	128	128	64
R	1	32	64	128	128	128	128	64
R	0	64	64	128	128	128	128	128
R	0	128	256	128	256	256	128	256
R	1	32	64	128	128	128	128	64
R	0	256	256	128	256	128	128	256
R	0	128	256	128	256	256	128	256
R	0	32	64	128	128	128	128	64
R	1	128	128	128	128	128	128	128
R	0	256	256	128	256	128	128	256
R	1	128	256	128	256	256	128	256
R	1	32	64	128	128	128	128	256
R	0	128	256	128	256	256	128	256
R	1	64	256	128	128	128	128	64
R	0	128	256	128	256	128	128	256
R	0	128	256	128	256	256	128	256
R	0	128	256	128	256	256	128	256
R	0	64	64	128	128	128	128	128
R	0	32	128	128	128	128	128	64
R	0	128	256	128	256	256	128	256
R	0	32	64	128	128	128	128	64
R	0	128	256	128	256	256	128	256
R	1	64	64	128	128	128	128	256
R	0	128	256	128	256	128	128	256
R	0	128	256	128	256	256	128	256

R	**0**	128	**256**	**128**	**256**	**256**	**128**	**256**
R	**1**	32	64	**128**	128	128	128	256
R	**1**	128	64	**128**	128	128	128	64
R	**1**	128	**256**	**128**	**256**	**128**	**128**	**256**
R	**0**	128	**256**	**128**	**256**	**256**	**128**	**256**
R	**0**	128	**256**	**128**	**256**	**256**	**128**	**256**
R	**0**	128	64	**128**	128	128	128	256
R	**0**	256	**256**	**128**	**256**	**128**	**128**	**256**
R	**0**	128	**256**	**128**	**256**	**256**	**128**	**256**
R	**0**	32	64	**128**	128	128	128	256
R	**0**	256	**256**	**128**	**256**	**128**	**128**	**256**
R	**0**	128	**256**	**128**	**256**	**256**	**128**	**256**
R	**1**	256	64	**128**	128	64	128	64
R	**1**	256	**256**	**128**	**256**	**128**	**128**	**256**
R	**0**	128	**256**	**128**	**256**	**256**	**128**	**256**
R	**1**	64	64	**128**	128	128	128	256
R	**0**	128	**256**	**128**	**256**	**256**	**128**	**256**
R	**0**	128	**256**	**128**	**256**	**256**	**128**	**256**
R	**0**	128	**256**	**128**	**256**	**256**	**128**	**256**
R	**1**	128	**256**	**128**	**256**	**256**	**128**	**256**
R	**1**	128	**256**	**128**	**256**	**256**	**128**	**256**
R	**0**	128	**256**	**128**	**256**	**256**	**128**	**256**
R	**0**	32	64	**128**	128	128	128	32
R	**0**	128	64	**128**	128	128	128	64
R	**0**	128	**256**	**128**	**256**	**256**	**128**	**256**
R	**0**	128	**256**	**128**	**256**	**256**	**128**	**256**
R	**0**	256	**256**	**128**	**128**	**256**	**128**	**256**
R	**0**	128	**256**	**128**	**256**	**256**	**128**	**256**
R	**1**	64	64	**128**	128	128	128	64
R	**1**	256	**256**	**128**	**256**	**128**	**128**	**256**
R	**1**	128	**256**	**128**	**256**	**256**	**128**	**256**
R	**0**	32	64	**128**	128	128	128	128
R	**0**	32	64	**128**	128	128	128	64
R	**1**	64	64	**128**	128	128	128	128
R	**1**	128	**256**	**128**	**256**	**256**	**128**	**256**
R	**0**	32	64	**128**	128	128	128	64
R	**0**	64	64	**128**	128	128	128	64
R	**0**	128	**256**	**128**	**256**	**256**	**128**	**256**
R	**1**	64	64	**128**	128	128	128	128
R	**0**	128	**256**	**128**	**256**	**256**	**128**	**256**
R	**0**	128	**256**	**128**	**256**	**256**	**128**	**256**
R	**0**	256	**256**	**128**	**128**	**256**	**128**	**256**
R	**1**	128	**256**	**128**	**256**	**256**	**128**	**256**
R	**1**	64	64	**128**	128	128	128	64
R	**1**	128	**256**	**128**	**256**	**256**	**128**	**256**
R	**1**	32	64	**128**	128	128	128	64
R	**0**	128	**256**	**128**	**256**	**256**	**128**	**256**
R	**1**	32	64	**128**	128	128	128	128
R	**0**	32	64	**128**	128	64	128	64
R	**0**	128	**256**	**128**	**256**	**256**	**128**	**256**

R	**0**	64	64	**128**	128	128	128	64
R	**0**	32	64	**128**	128	128	128	256
R	**0**	64	64	**128**	128	128	128	128
R	**1**	128	**256**	**128**	**256**	**256**	**128**	**256**
R	**1**	32	64	**128**	128	128	128	256
R	**0**	256	**256**	**128**	**256**	**128**	**128**	**256**
R	**0**	128	**256**	**128**	**256**	**256**	**128**	**256**
R	**1**	64	64	**128**	128	128	128	64
R	**0**	256	**256**	**128**	**256**	**128**	**128**	**256**
R	**0**	128	**256**	**128**	**256**	**256**	**128**	**256**
R	**1**	32	64	**128**	128	128	128	256
R	**1**	32	64	**128**	128	128	128	64
R	**0**	128	**256**	**128**	**256**	**256**	**128**	**256**
R	**0**	128	**256**	**128**	**256**	**256**	**128**	**256**
R	**0**	256	**256**	**128**	**128**	**256**	**128**	**256**
R	**1**	128	**256**	**128**	**256**	**256**	**128**	**256**
R	**1**	128	**256**	**128**	**256**	**256**	**128**	**256**
R	**0**	32	64	**128**	128	128	128	256
R	**0**	128	64	**128**	128	128	128	64
R	**1**	64	64	**128**	128	128	128	128
R	**0**	128	**256**	**128**	**256**	**128**	**128**	**256**
R	**0**	128	**256**	**128**	**256**	**256**	**128**	**256**
R	**0**	128	**256**	**128**	**256**	**256**	**128**	**256**
R	**0**	32	64	**128**	128	128	128	64
R	**1**	64	64	**128**	128	128	128	128
S	**1**	32	64	**128**	128	128	128	64
R	**0**	128	**256**	**128**	**256**	**256**	**128**	**256**
R	**0**	128	**256**	**128**	**256**	**256**	**128**	**256**
R	**1**	256	**256**	**128**	**128**	**256**	**128**	**256**
R	**0**	128	**256**	**128**	**256**	**256**	**128**	**256**
R	**0**	128	**256**	**128**	**256**	**256**	**128**	**256**
R	**1**	64	64	**128**	128	128	128	128
R	**0**	256	**256**	**128**	**256**	**128**	**128**	**256**
R	**0**	128	**256**	**128**	**256**	**256**	**128**	**256**
R	**1**	128	64	**128**	128	128	128	128
R	**0**	256	**256**	**128**	**256**	**128**	**128**	**256**
R	**1**	128	**256**	**128**	**256**	**256**	**128**	**256**
R	**1**	256	64	**128**	128	128	128	64
R	**0**	128	**256**	**128**	**256**	**256**	**128**	**256**
R	**1**	256	64	**128**	128	128	128	64
R	**0**	256	**256**	**128**	**256**	**128**	**128**	**256**
R	**0**	128	**256**	**128**	**256**	**256**	**128**	**256**
R	**0**	32	64	**128**	128	128	128	256
R	**0**	128	**256**	**128**	**256**	**256**	**128**	**256**
R	**0**	128	**256**	**128**	**256**	**256**	**128**	**256**
R	**1**	128	**256**	**128**	**256**	**256**	**128**	**256**
R	**1**	256	**256**	**128**	**128**	**256**	**128**	**256**
R	**0**	128	**256**	**128**	**256**	**256**	**128**	**256**
R	**0**	128	**256**	**128**	**256**	**256**	**128**	**256**
R	**0**	128	64	**128**	128	128	128	128

R	**0**	32	64	**128**	128	128	128	64
R	**0**	128	**256**	**128**	**256**	**256**	**128**	**256**
R	**0**	128	**256**	**128**	**256**	**256**	**128**	**256**
R	**0**	256	**256**	**128**	**128**	**256**	**128**	**256**
R	**1**	128	**256**	**128**	**256**	**256**	**128**	**256**
R	**0**	256	64	**128**	128	128	128	128
R	**0**	128	**256**	**128**	**256**	**256**	**128**	**256**
R	**1**	128	**256**	**128**	**256**	**256**	**128**	**256**
R	**0**	256	**256**	**128**	**128**	**256**	**128**	**256**
R	**0**	128	**256**	**128**	**256**	**256**	**128**	**256**
R	**1**	32	64	**128**	128	128	128	64
R	**0**	128	**256**	**128**	**256**	**256**	**128**	**256**
R	**0**	128	**256**	**128**	**256**	**256**	**128**	**256**
R	**1**	128	**256**	**128**	**256**	**256**	**128**	**256**
R	**0**	128	**256**	**128**	**256**	**256**	**128**	**256**
R	**0**	128	**256**	**128**	**256**	**256**	**128**	**256**
R	**1**	128	**256**	**128**	**256**	**256**	**128**	**256**
R	**0**	128	**256**	**128**	**256**	**256**	**128**	**256**
R	**0**	128	64	**128**	128	128	128	128
R	**0**	256	**256**	**128**	**256**	**128**	**128**	**256**
R	**1**	128	**256**	**128**	**256**	**256**	**128**	**256**
R	**0**	128	**256**	**128**	**256**	**256**	**128**	**256**
R	**1**	32	64	**128**	128	128	128	128
R	**1**	256	**256**	**128**	**256**	**128**	**128**	**256**
R	**0**	128	**256**	**128**	**256**	**256**	**128**	**256**
S	**1**	256	64	**128**	128	128	128	64
R	**0**	32	64	**128**	128	128	128	128
R	**0**	32	64	**128**	128	128	128	64
R	**0**	128	**256**	**128**	**256**	**256**	**128**	**256**
R	**1**	128	**256**	**128**	**256**	**256**	**128**	**256**
R	**0**	256	**256**	**128**	**128**	**256**	**128**	**256**
R	**0**	128	**256**	**128**	**256**	**256**	**128**	**256**
R	**1**	128	64	**128**	128	128	128	128
R	**1**	128	**256**	**128**	**256**	**256**	**128**	**256**
R	**0**	32	64	**128**	128	128	128	64
R	**0**	128	**256**	**128**	**256**	**256**	**128**	**256**
R	**0**	64	64	**128**	128	128	128	128
S	**1**	32	64	**128**	128	128	128	64
R	**0**	32	64	**128**	128	128	128	64
R	**1**	64	64	**128**	128	128	128	64
R	**1**	128	**256**	**128**	**256**	**256**	**128**	**256**
S	**0**	128	64	**128**	128	128	128	256
R	**1**	256	**256**	**128**	**256**	**128**	**128**	**256**
R	**0**	128	**256**	**128**	**256**	**256**	**128**	**256**
S	**0**	128	64	**128**	128	128	128	64
R	**0**	256	**256**	**128**	**256**	**128**	**128**	**256**
R	**0**	128	**256**	**128**	**256**	**256**	**128**	**256**
S	**0**	32	64	**128**	128	128	128	128
R	**0**	128	**256**	**128**	**256**	**256**	**128**	**256**
S	**0**	256	64	**128**	128	128	128	64

S	**0**	128	**256**	**128**	**256**	**256**	**128**	**256**
S	**1**	64	64	**128**	128	64	128	64
S	***1***	64	64	**128**	128	128	128	128
R	**0**	128	**256**	**128**	**256**	**256**	**128**	**256**
S	**1**	128	32	**128**	128	128	64	64
