# Supplementary material for: Haematological Profile and Antibiotic Resistance of Bacteria Responsible for Enteric Infections Isolated From Patients Suffering From Malaria and Enteric Infections on Consultation at the Dschang Regional Hospital
Source: Can J Infect Dis Med Microbiol. 2024 Oct 25;2024:3383995. doi: 10.1155/2024/3383995 (PMC11530289; doi:10.1155/2024/3383995)
Supplement: Supporting Information — Additional supporting information can be found online in the Supporting Information section. [file 3383995.f1.zip › Supporting_file (1).docx]

Haematological profile and antibiotic resistance of bacteria responsible for Enteric Infections isolated from patients suffering from Malaria and Enteric Infections on consultation at the Dschang Regional Hospital

Roland Y. Ngai, Wiliane J.T. Marbou, Armelle T. Mbaveng*, Victor Kuete**

^1^Department of Biochemistry, Faculty of Science, University of Dschang, Cameroon

*Corresponding author:*

**E-mail: armbatsa@yahoo.fr (Prof. Dr. Armelle T. Mbaveng)*

***E-mail:* [*kuetevictor@yahoo.fr*](mailto:kuetevictor@yahoo.fr) *(Prof. Dr. Victor Kuete)*

*Authors e-mail:*

*Ngai R. Yinkfu:* [*ngairolandidi@yahoo.com*](mailto:ngairolandidi@yahoo.com)

*Wiliane J.T. Marbou:* [*marboutakougoum@yahoo.fr*](mailto:marboutakougoum@yahoo.fr)

**Table S1**. Antibiotics used and susceptibility classification standards.

| **Mechanism of action** | **Family of antibiotic** | **Antibiotic name** | **abbreviation** | **Concentratio of antibiotic disc (µg)** | **Critical diameter of antibiotic disc**  **(mm)** | | |
| --- | --- | --- | --- | --- | --- | --- | --- |
|  |  |  |  |  | **S** | **I** | **R** |
| Inhibition of synthesis of bacteria envelop | Beta-Lactamines | Ceftazidime  Amoxicillin + clavulanic acid  Amoxicillin  Penicillin G | CAZ  AMC  AMOX  PEN | 30  20/10  10  10 | \|  \| \| --- \|   ≥26  ≥18  ≥15  ≥13 | \|  \| \| --- \|   21<∅≤26  14≤∅≤17  13≤∅≤14  11≤∅≤12 | \|  \| \| --- \|   <21  ≤13  ≤12  ≤10 |
| Inhibition of protein synthesis | Aminosides  Cyclines  Phenicol  Macrolides | Amikacin  Doxycycline  Chloramphenicol  Clindamycin  Erythromycin | AKI  DOX  CHL  CDA  ERY | 30  30  25  10  15 | ≥18  ≥19 ≥17  ≥14  ≥22 | 15≤∅≤16  11<∅≤13 13≤∅≤17  11<∅≤13  17<∅≤22 | ≥18  ≤10  ≤12 <10  <21 |
| Inhibition of synthesis of nucleic acids | Quinolones | Ciprofloxacin  Levofloxacin | CIP  LEV | 5  5 | 25  23 | 22<∅≤24  - | <22  19 |
| Inhibition of synthesis of Folic acid | Sulfamides | Trimethoprim/sulfamethoxazol  Cotrimoxazole | SXT  COT | 25 | ≥16  ≥14 | \|  \| \| --- \|   11≤∅≤15  11<∅≤14 | ≤10  <11 |

S: sensitive; I: intermediary; R: resistant; ∅: diameter of inhibition zone

**Table S2.** Cut-off presented by different bacteria species

| **Bacteria species** | **Cut-off (µg/mL)** | | | | | | | **Reference** |
| --- | --- | --- | --- | --- | --- | --- | --- | --- |
|  | Outstanding activity | Excellent activity | Very good activity | Good activity | Average activity | Weak activity | No activity |  |
| *E. coli*  *Enterobacter aerogenes*  *Enterobacter cloacae*  *Klebsiella pneumonia* | MIC≤8 | 8<MIC≤64 | 64<MIC≤128 | 128<MIC≤256 | 256<MIC≤512 | 512<MIC≤1024 | MIC>1024 | [1] |
| *Pseudomonas aeruginosa,* | MIC≤32 | 32<MIC≤128 | 128<MIC≤256 | 256<MIC≤512 | 512<MIC≤1024 | MIC>1024 | MIC>1024 | [2] |
| Gram positive bacteria | MIC≤8 | 8<MIC≤40 | 40<MIC≤128 | 128<MIC≤320 | 320<MIC≤625 | 625<MIC≤1024 | MIC>1024. | [3] |

**Table S3.** Culture media and Antimicrobials

| Culture media | Weight of powder (g) | Volume of distilled water (ml) | Sterilization time in the autoclave (min) at 121^o^C |
| --- | --- | --- | --- |
| EMB | 36 | 1000 | 15 |
| CHAPMAN | 111 | 1000 | 15 |
| MHA | 38 | 1000 | 15 |
| MHB | 26 | 1000 | 15 |
| SS | 36 | 1000 | / |
| MACONKEY | 50.03 | 1000 | 15 |
| HECTOEN | 34 | 1000 | / |

| **Antimalarial** | **Quantity** |
| --- | --- |
| Quinine | 600 mg |
| Artemethe | (80 mg) |
| G.cospe | 500/25 mg |
| Artesunate amodiaquine | 100/270 mg |
| Maloox | 25/500mg |
| Nemether | 320/40 mg |
| Suriquine | 250mg |

**References**

[1] V. Kuete, "Ethnopharmacology, phytochemistry and pharmacology of potent antibacterial medicinal plants from Africa, *Advances in Botanical Research"*, pp. 353-660, 2023.

[2] S.B. Tankeo, V. Kuete, "Chapter Seven - African plants acting on Pseudomonas aeruginosa: Cut-off points for the antipseudomonal agents from plants", *Advances in Botanical Research*, pp. 337-412, 2023.

[3] B.E.N. Wamba, A.T. Mbaveng, V. Kuete, "Chapter Eight - Fighting Gram-positive bacteria with African medicinal plants: Cut-off values for the classification of the activity of natural products", *Advances in Botanical Research*, Academic Press, pp. 413-522, 2023.
